# Supplementary material for: Do generative models learn rare generative factors?
Source: Front Artif Intell. 2025 Nov 19;8:1697139. doi: 10.3389/frai.2025.1697139 (PMC12679468; doi:10.3389/frai.2025.1697139)
Supplement: Supplementary file 1 [file Data_Sheet_1.pdf]

# Supplementary Material

## 1 MODELS ARCHITECTURES

This appendix details the architectures and training procedures for the oracle classifiers, GAN, VAE, and DM.

### 1.1 Oracle Classifiers Architecture

#### 1.1.1 Digit Classifier

- Conv2d(3, 10, kernel_size=(5, 5), stride=(1, 1))
- F.max_pool2d(..., 2) applies 2x2 max
- Relu
- (conv2): Conv2d(10, 20, kernel_size=(5, 5), stride=(1, 1))
- (conv2_drop): Dropout2d(p=0.5, inplace=False)
- F.max_pool2d(..., 2) applies 2x2 max
- Relu()
- (fc1): Linear(in_features=3380, out_features=50, bias=True)
- Relu()
- Dropout(input, p=0.5, training=True, inplace=False)
- (fc2): Linear(in_features=50, out_features=10, bias=True)
- Softmax()

#### 1.1.2 Generative Factor Classifier

- Conv2d(3, 10, kernel_size=(5, 5), stride=(1, 1))
- F.max_pool2d(..., 2) applies 2x2 max
- Relu()
- (conv2): Conv2d(10, 20, kernel_size=(5, 5), stride=(1, 1))
- (conv2_drop): Dropout2d(p=0.5, inplace=False)
- F.max_pool2d(..., 2) applies 2x2 max
- Relu()
- (fc1): Linear(in_features=3380, out_features=50, bias=True)
- Relu ()
- Dropout(input, p=0.5, training=True, inplace=False)
- (fc2): Linear(in_features=50, out_features=1, bias=True)
- Sigmoid()

### 1.2 Diffusion Model

#### 1.2.1 U-Net Architecture

- **Input**: RGB images of size $64 \times 64$ (3 input channels).
- **Down Block 1**:
- Conv2d(3, 128, kernel_size=(3, 3), stride=(1, 1), padding=(1, 1), bias=True)
- ReLU(inplace=True)

- Conv2d(128, 128, kernel_size=(3, 3), stride=(1, 1), padding=(1, 1), bias=True)
- ReLU(inplace=True)
- **Down Block 2**:
- Conv2d(128, 128, kernel_size=(3, 3), stride=(2, 2), padding=(1, 1), bias=True)
- ReLU(inplace=True)
- Conv2d(128, 128, kernel_size=(3, 3), stride=(1, 1), padding=(1, 1), bias=True)
- ReLU(inplace=True)
- **Down Block 3**:
- Conv2d(128, 256, kernel_size=(3, 3), stride=(2, 2), padding=(1, 1), bias=True)
- ReLU(inplace=True)
- Conv2d(256, 256, kernel_size=(3, 3), stride=(1, 1), padding=(1, 1), bias=True)
- ReLU(inplace=True)
- **Down Block 4**:
- Conv2d(256, 256, kernel_size=(3, 3), stride=(2, 2), padding=(1, 1), bias=True)
- ReLU(inplace=True)
- Conv2d(256, 256, kernel_size=(3, 3), stride=(1, 1), padding=(1, 1), bias=True)
- ReLU(inplace=True)
- **Down Block 5 (with Attention)**:
- Conv2d(256, 512, kernel_size=(3, 3), stride=(2, 2), padding=(1, 1), bias=True)
- Self-Attention Layer
- Conv2d(512, 512, kernel_size=(3, 3), stride=(1, 1), padding=(1, 1), bias=True)
- ReLU(inplace=True)
- **Down Block 6**:
- Conv2d(512, 512, kernel_size=(3, 3), stride=(2, 2), padding=(1, 1), bias=True)
- ReLU(inplace=True)
- **Bottleneck**:
- Conv2d(512, 512, kernel_size=(3, 3), stride=(1, 1), padding=(1, 1), bias=True)
- ReLU(inplace=True)
- **Up Block 1**:
- ConvTranspose2d(512, 512, kernel_size=(3, 3), stride=(2, 2), padding=(1, 1), bias=True)
- ReLU(inplace=True)
- **Up Block 2 (with Attention)**:
- Self-Attention Layer
- ConvTranspose2d(512, 256, kernel_size=(3, 3), stride=(2, 2), padding=(1, 1), bias=True)
- ReLU(inplace=True)
- **Up Block 3**:
- ConvTranspose2d(256, 256, kernel_size=(3, 3), stride=(2, 2), padding=(1, 1), bias=True)
- ReLU(inplace=True)
- **Up Block 4**:
- ConvTranspose2d(256, 256, kernel_size=(3, 3), stride=(2, 2), padding=(1, 1), bias=True)
- ReLU(inplace=True)
- **Up Block 5**:
- ConvTranspose2d(256, 128, kernel_size=(3, 3), stride=(2, 2), padding=(1, 1), bias=True)
- ReLU(inplace=True)
- **Up Block 6**:

- ConvTranspose2d(128, 128, kernel_size=(3, 3), stride=(2, 2), padding=(1, 1), bias=True)
- ReLU(inplace=True)
- **Output Layer**:
- Conv2d(128, 3, kernel_size=(3, 3), stride=(1, 1), padding=(1, 1), bias=True)

## 1.3 GAN

### 1.3.1 Generator

- ConvTranspose2d(5, 512, kernel_size=(4, 4), stride=(1, 1), bias=False)
- BatchNorm2d(512, eps=1e-05, momentum=0.1, affine=True, track_running_stats=True)
- ReLU(inplace=True)
- ConvTranspose2d(512, 256, kernel_size=(4, 4), stride=(2, 2), padding=(1, 1), bias=False)
- BatchNorm2d(256, eps=1e-05, momentum=0.1, affine=True, track_running_stats=True)
- ReLU(inplace=True)
- ConvTranspose2d(256, 128, kernel_size=(4, 4), stride=(2, 2), padding=(1, 1), bias=False)
- BatchNorm2d(128, eps=1e-05, momentum=0.1, affine=True, track_running_stats=True)
- ReLU(inplace=True)
- ConvTranspose2d(128, 64, kernel_size=(4, 4), stride=(2, 2), padding=(1, 1), bias=False)
- BatchNorm2d(64, eps=1e-05, momentum=0.1, affine=True, track_running_stats=True)
- ReLU(inplace=True)
- ConvTranspose2d(64, 3, kernel_size=(4, 4), stride=(2, 2), padding=(1, 1), bias=False)
- Tanh()

### 1.3.2 Discriminator

- Conv2d(3, 64, kernel_size=(4, 4), stride=(2, 2), padding=(1, 1), bias=False)
- LeakyReLU(negative_slope=0.2, inplace=True)
- Conv2d(64, 128, kernel_size=(4, 4), stride=(2, 2), padding=(1, 1), bias=False)
- BatchNorm2d(128, eps=1e-05, momentum=0.1, affine=True, track_running_stats=True)
- LeakyReLU(negative_slope=0.2, inplace=True)
- Conv2d(128, 256, kernel_size=(4, 4), stride=(2, 2), padding=(1, 1), bias=False)
- BatchNorm2d(256, eps=1e-05, momentum=0.1, affine=True, track_running_stats=True)
- LeakyReLU(negative_slope=0.2, inplace=True)
- Conv2d(256, 512, kernel_size=(4, 4), stride=(2, 2), padding=(1, 1), bias=False)
- BatchNorm2d(512, eps=1e-05, momentum=0.1, affine=True, track_running_stats=True)
- LeakyReLU(negative_slope=0.2, inplace=True)
- Conv2d(512, 1, kernel_size=(4, 4), stride=(1, 1), bias=False)
- Sigmoid()

### 1.4 VAE

#### 1.4.1 Encoder

- Conv2d(3, 32, kernel_size=(4, 4), stride=(2, 2), padding=(1, 1))
- ReLU()
- Conv2d(32, 64, kernel_size=(4, 4), stride=(2, 2), padding=(1, 1))
- ReLU()
- Conv2d(64, 128, kernel_size=(4, 4), stride=(2, 2), padding=(1, 1))
- ReLU()
- Conv2d(128, 256, kernel_size=(4, 4), stride=(2, 2), padding=(1, 1))
- ReLU()
- (fc1):Linear(in_features=4096, out_features=5, bias=True)
- (fc2):Linear(in_features=4096, out_features=5, bias=True)

#### 1.4.2 Decoder

- ConvTranspose2d(256, 128, kernel_size=(4, 4), stride=(2, 2), padding=(1, 1))
- ReLU()
- ConvTranspose2d(128, 64, kernel_size=(4, 4), stride=(2, 2), padding=(1, 1))
- ReLU()
- ConvTranspose2d(64, 32, kernel_size=(4, 4), stride=(2, 2), padding=(1, 1))
- ReLU()
- ConvTranspose2d(32, 3, kernel_size=(4, 4), stride=(2, 2), padding=(1, 1))
- Sigmoid()

### 1.5 Training details

The GANs and VAEs are trained for 200 epochs on 3x64x64 images with a batch size of 128. For MNIST-derived datasets, we use a latent vector size of 5, while for CompCars, we increase this to 20. GAN model selection is based on visual inspection and Gradient Magnitude Similarity Deviation (GMSD) (**?**), while VAE selection uses validation loss. GAN-specific parameters: learning rate: 0.0002, Adam optimizer with $\beta_1 = 0.5$. VAE-specific parameters: learning rate: 0.001, Adam optimizer with $\beta_1 = 0.9$.

For DM, the training details are as follows:

- **Diffusion Scheduler**: A `DDPMScheduler` is used to guide the training, with 1000 timesteps to progressively add noise and learn the denoising process.
- **Loss Function**: Mean Squared Error (MSE) loss is used for pixel-wise comparison between the model's output and the ground truth.
- **Optimizer**: The Adam optimizer is used with a learning rate of $10^{-4}$.

## 2 ORACLE CLASSIFIERS DATA AND RESULTS

Table S1 shows the test-set accuracy of oracle classifiers. Some images from training data are shown in Figure S1.

Table S2 shows the subset of CompCars dataset we employed.

**Table S1.** Oracle Classifiers Test-set Accuracy

| MNIST dataset | | |
|---|---|---|
| **Factor** | **Digit classifier** | **Factor classifier** |
| Colour | 97% | 100% |
| Fracture | 94% | 94% |
| Swell | 96% | 95% |
| Thick | 95% | 96% |
| Thin | 95% | 98% |
| **CompCars dataset** | | |
| | **Car Make classifier** | **Factor (Colour) classifier** |
| Colour | 92% | 99% |

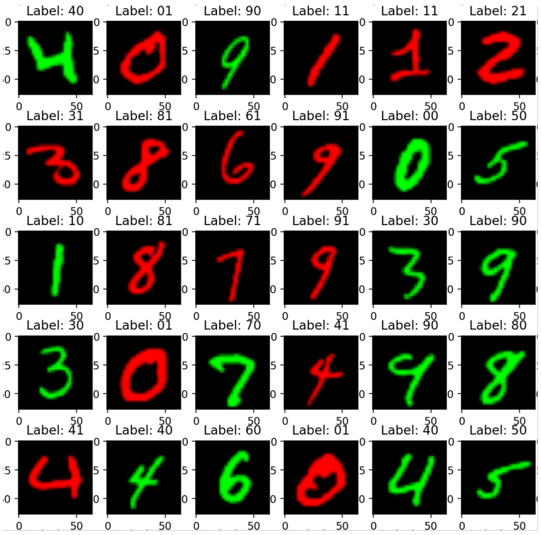


Figure S1: An example of the dataset used for colour oracle classifiers training.

**Table S2.** Subset of CompCars dataset used for oracle classifiers training.

| Make | Color | Count |
|---|---|---|
| Volkswagen | black | 362 |
| Toyota | black | 363 |
| Volkswagen | white | 361 |
| Toyota | white | 362 |

# 3 RESULTS FOR TRAINING ON BALANCED DATASETS

The results for training on balanced datasets $P_c^{(u)}$ are reported in Table S3 and Table S4 for the MNIST and CompCars datasets, respectively.

# 4 RARE DATA AND EXTREME CASE

Some examples of rare data are shown in Figure S2. Next, we report results using GAN, VAE and DM.

## 4.1 GAN Results (visual insight)

We provide visual insight into our results to visually inspect the images generated by GAN.

Figure S9 displays images generated by the GAN trained on data without RGFs.

**Table S3.** MNIST: Generative factor proportions $P_c^{(u)}$ for VAE, GAN and DM.

| Digit | Generative Factor Proportions $P_c^{(u)}$ | | | | | | | | | | | | | | |
|---|---|---|---|---|---|---|---|---|---|---|---|---|---|---|---|
| | **Colour** | | | **Fracture** | | | **Swell** | | | **Thick** | | | **Thin** | | |
| | **red** | **green** | $P_c^{(u)}$ | **no** | **yes** | $P_c^{(u)}$ | **no** | **yes** | $P_c^{(u)}$ | **no** | **yes** | $P_c^{(u)}$ | **no** | **yes** | $P_c^{(u)}$ |
| **VAE** | | | | | | | | | | | | | | | |
| 0 | 40 | 35 | 0.47 | 51 | 24 | 0.32 | 65 | 17 | 0.21 | 49 | 27 | 0.36 | 36 | 56 | 0.61 |
| 1 | 33 | 37 | 0.53 | 45 | 17 | 0.27 | 37 | 67 | 0.64 | 54 | 30 | 0.36 | 21 | 48 | 0.70 |
| 2 | 71 | 67 | 0.49 | 66 | 33 | 0.33 | 53 | 32 | 0.38 | 74 | 46 | 0.38 | 59 | 75 | 0.56 |
| 3 | 62 | 46 | 0.43 | 67 | 40 | 0.37 | 72 | 23 | 0.24 | 33 | 26 | 0.44 | 44 | 43 | 0.49 |
| 4 | 57 | 52 | 0.48 | 64 | 47 | 0.42 | 64 | 53 | 0.45 | 86 | 57 | 0.40 | 43 | 65 | 0.60 |
| 5 | 62 | 71 | 0.53 | 46 | 44 | 0.49 | 94 | 15 | 0.14 | 65 | 43 | 0.40 | 24 | 54 | 0.69 |
| 6 | 49 | 52 | 0.51 | 86 | 44 | 0.34 | 88 | 46 | 0.34 | 74 | 59 | 0.44 | 37 | 74 | 0.67 |
| 7 | 62 | 48 | 0.44 | 70 | 29 | 0.29 | 46 | 52 | 0.53 | 61 | 49 | 0.45 | 51 | 50 | 0.50 |
| 8 | 35 | 40 | 0.53 | 96 | 17 | 0.15 | 49 | 41 | 0.46 | 46 | 45 | 0.49 | 52 | 61 | 0.54 |
| 9 | 34 | 47 | 0.58 | 79 | 35 | 0.31 | 42 | 44 | 0.51 | 44 | 32 | 0.42 | 46 | 61 | 0.57 |
| Total | 505 | 495 | 0.50 | 670 | 330 | 0.33 | 610 | 390 | 0.39 | 586 | 414 | 0.41 | 413 | 587 | 0.59 |
| **GAN** | | | | | | | | | | | | | | | |
| 0 | 53 | 88 | 0.62 | 110 | 16 | 0.13 | 51 | 62 | 0.55 | 71 | 24 | 0.25 | 54 | 46 | 0.46 |
| 1 | 72 | 38 | 0.35 | 25 | 79 | 0.76 | 65 | 73 | 0.53 | 85 | 58 | 0.41 | 53 | 50 | 0.49 |
| 2 | 48 | 36 | 0.43 | 27 | 37 | 0.58 | 32 | 48 | 0.60 | 93 | 19 | 0.17 | 51 | 43 | 0.46 |
| 3 | 55 | 41 | 0.43 | 50 | 68 | 0.58 | 46 | 60 | 0.57 | 51 | 50 | 0.50 | 35 | 58 | 0.62 |
| 4 | 45 | 42 | 0.48 | 46 | 63 | 0.58 | 31 | 38 | 0.55 | 26 | 33 | 0.56 | 48 | 81 | 0.63 |
| 5 | 53 | 42 | 0.44 | 36 | 49 | 0.58 | 27 | 55 | 0.67 | 31 | 33 | 0.52 | 22 | 65 | 0.75 |
| 6 | 69 | 47 | 0.41 | 39 | 56 | 0.59 | 36 | 70 | 0.66 | 24 | 127 | 0.84 | 33 | 90 | 0.73 |
| 7 | 67 | 47 | 0.41 | 62 | 47 | 0.43 | 54 | 52 | 0.49 | 82 | 55 | 0.40 | 35 | 63 | 0.64 |
| 8 | 17 | 52 | 0.75 | 79 | 42 | 0.35 | 25 | 59 | 0.70 | 38 | 38 | 0.50 | 40 | 49 | 0.55 |
| 9 | 35 | 53 | 0.60 | 38 | 31 | 0.45 | 54 | 62 | 0.53 | 31 | 31 | 0.50 | 44 | 40 | 0.48 |
| Total | 514 | 486 | 0.49 | 512 | 488 | 0.49 | 421 | 579 | 0.58 | 532 | 468 | 0.47 | 415 | 585 | 0.59 |
| **Diffusion Models** | | | | | | | | | | | | | | | |
| 0 | 64 | 48 | 0.43 | 72 | 49 | 0.40 | 82 | 36 | 0.31 | 72 | 20 | 0.22 | 38 | 88 | 0.70 |
| 1 | 104 | 51 | 0.32 | 70 | 52 | 0.43 | 66 | 64 | 0.49 | 78 | 54 | 0.41 | 52 | 63 | 0.55 |
| 2 | 29 | 71 | 0.71 | 63 | 62 | 0.5 | 45 | 48 | 0.52 | 41 | 30 | 0.42 | 37 | 99 | 0.73 |
| 3 | 31 | 41 | 0.57 | 26 | 41 | 0.61 | 35 | 48 | 0.58 | 39 | 34 | 0.47 | 26 | 50 | 0.66 |
| 4 | 32 | 57 | 0.64 | 39 | 50 | 0.56 | 48 | 44 | 0.48 | 55 | 30 | 0.35 | 56 | 55 | 0.50 |
| 5 | 45 | 46 | 0.51 | 48 | 56 | 0.54 | 40 | 30 | 0.43 | 82 | 29 | 0.26 | 22 | 68 | 0.76 |
| 6 | 47 | 66 | 0.58 | 61 | 38 | 0.38 | 58 | 54 | 0.48 | 69 | 57 | 0.45 | 35 | 55 | 0.61 |
| 7 | 63 | 33 | 0.34 | 61 | 36 | 0.37 | 32 | 66 | 0.67 | 62 | 56 | 0.47 | 33 | 61 | 0.65 |
| 8 | 58 | 63 | 0.52 | 98 | 34 | 0.26 | 81 | 78 | 0.49 | 54 | 75 | 0.58 | 65 | 38 | 0.37 |
| 9 | 29 | 22 | 0.43 | 29 | 15 | 0.34 | 17 | 28 | 0.62 | 9 | 54 | 0.86 | 23 | 36 | 0.61 |
| Total | 502 | 498 | 0.50 | 567 | 433 | 0.43 | 504 | 496 | 0.50 | 561 | 439 | 0.44 | 387 | 613 | 0.61 |

**Table S4.** CompCars: Generative factor proportions $P_c^{(u)}$ for VAE, GAN, and DM.

| Make | VAE | | | GAN | | | Diffusion Models | | |
|---|---|---|---|---|---|---|---|---|---|
| | **Black** | **White** | $P_c^{(u)}$ | **Black** | **White** | $P_c^{(u)}$ | **Black** | **White** | $P_c^{(u)}$ |
| **Volkswagen** | 254 | 219 | 0.46 | 226 | 238 | 0.51 | 296 | 133 | 0.31 |
| **Toyota** | 277 | 250 | 0.47 | 337 | 199 | 0.37 | 438 | 133 | 0.23 |
| **Total** | 531 | 469 | 0.47 | 563 | 437 | 0.44 | 734 | 266 | 0.27 |

To illustrate GAN-generated images when trained with RGFs, we focus on specific digits and RGFs. Figures S10, S11, S12, and S13 show results for fractured-RGF (digit “2”), swell-RGF (digit “1”), Thick-RGF (digit ”2”), and Thin-RGF (digit “1”), respectively. These results demonstrate RGF memorization in skewed data. Additionally, Figures S14 and S15 present images generated using the spectral decoupling (SD) method, showing reduced memorization.

## 4.2 VAE results

We used the balanced datasets $D_u$ for each RGF, trained a VAE, and subsequently generated $M = 1000$ synthetic images. The resulting proportions of images, $P_c^{(u)}$, that exhibit the generative factor across each digit $c \in \{0, ..., 9\}$ are shown in Table S3.

The proportions $P_c^{(u)}$ and $P_c^{(r)}$, as illustrated in Figures S16, indicate VAEs memorize rare generative factors. For example, there’s a clear tendency to associate the color green with the digit ”2,” while the color

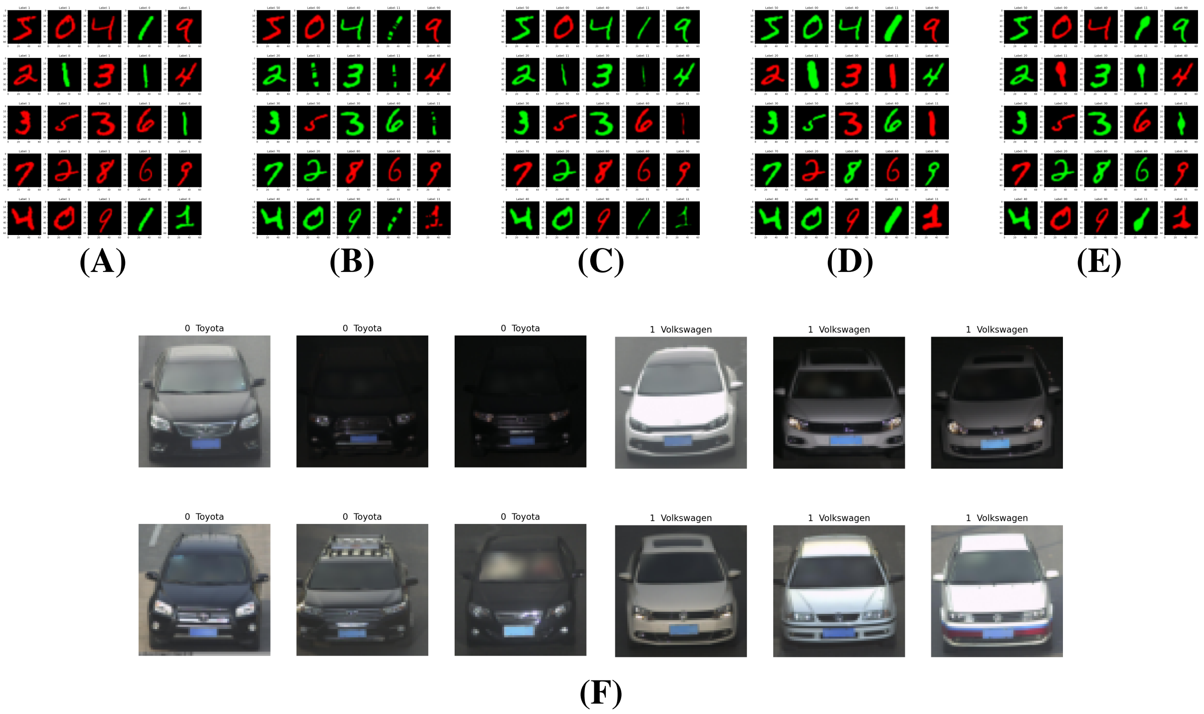

Figure S2: Examples of training images with different relevant generative factors (RGFs). (A) Colour as RGF. (B) Fracture as RGF. (C) Thin as RGF. (D) Thick as RGF. (E) Swell as RGF. (F) Colour as RGF in cars: Volkswagen in white and Toyota in black.

**Table S5.** GAN: z-scores (all images of digit "1" have RGF). Bold: similar proportions ($p > 0.05$), indicating RGF learning (Without spectral decoupling).

| Digit | Colour | | | Fracture | | | Swell | | | Thick | | | Thin | | |
|---|---|---|---|---|---|---|---|---|---|---|---|---|---|---|---|
| | red | green | z | no | yes | z | no | yes | z | no | yes | z | no | yes | z |
| 0 | 97 | 0 | - | 100 | 15 | **0.01** | 58 | 26 | -4.77 | 83 | 4 | -9.09 | 78 | 13 | -8.65 |
| 1 | 15 | 103 | 17.05 | 31 | 79 | **-0.97** | 28 | 72 | 4.23 | 51 | 62 | 2.96 | 8 | 131 | 22.90 |
| 2 | 96 | 1 | -40.92 | 67 | 6 | -15.49 | 64 | 37 | -4.87 | 58 | 3 | -4.36 | 59 | 14 | -5.82 |
| 3 | 88 | 1 | -37.48 | 86 | 20 | -10.30 | 61 | 24 | -5.89 | 52 | 8 | -8.36 | 137 | 18 | -19.58 |
| 4 | 105 | 0 | - | 90 | 11 | -15.20 | 78 | 8 | -14.59 | 101 | 1 | -56.40 | 43 | 9 | -8.71 |
| 5 | 91 | 0 | - | 61 | 36 | -4.26 | 64 | 18 | -9.86 | 85 | 5 | -19.24 | 66 | 17 | -12.31 |
| 6 | 132 | 0 | - | 103 | 3 | -34.87 | 106 | 25 | -13.66 | 123 | 6 | -42.80 | 96 | 35 | -11.97 |
| 7 | 99 | 1 | -40.20 | 99 | 6 | -16.46 | 91 | 22 | -7.93 | 106 | 16 | -8.80 | 78 | 33 | -7.90 |
| 8 | 87 | 1 | -65.37 | 63 | 28 | **-0.87** | 66 | 31 | -8.03 | 110 | 13 | -14.22 | 64 | 8 | -11.85 |
| 9 | 83 | 0 | - | 86 | 10 | -11.09 | 104 | 17 | -12.33 | 108 | 5 | -23.56 | 83 | 10 | -11.60 |
| Total | 893 | 107 | -39.18 | 786 | 214 | -21.28 | 720 | 280 | -21.13 | 877 | 123 | -33.41 | 712 | 288 | -21.09 |

red is more commonly linked with other digits. Specifically, when the digit "2" is assigned the green color, $93\%$ of generated images exhibit this trait, which is significantly different from the $52\%$ observed in Table S3. Conversely, the presence of green in images of other digits is minimal, hovering around $10\%$, indicating clear memorization of the green colour for digit "2" without extending this rare factor to other digits. A similar trend is evident when the colour factor is applied to digit "1". This tendency is further quantified by the z-scores presented in Tables S9 and S10 (corresponding to Figure S16), calculated according to Eq. 1. The large z-scores highlight significant differences in proportions between $P_c^{(u)}$ and $P_c^{(r)}$, confirming the memorization effect. This pattern of memorization also applies to other generative factors, although to a lesser degree. It indicates a broader tendency among VAEs to prioritize memorization over learning RGFs.

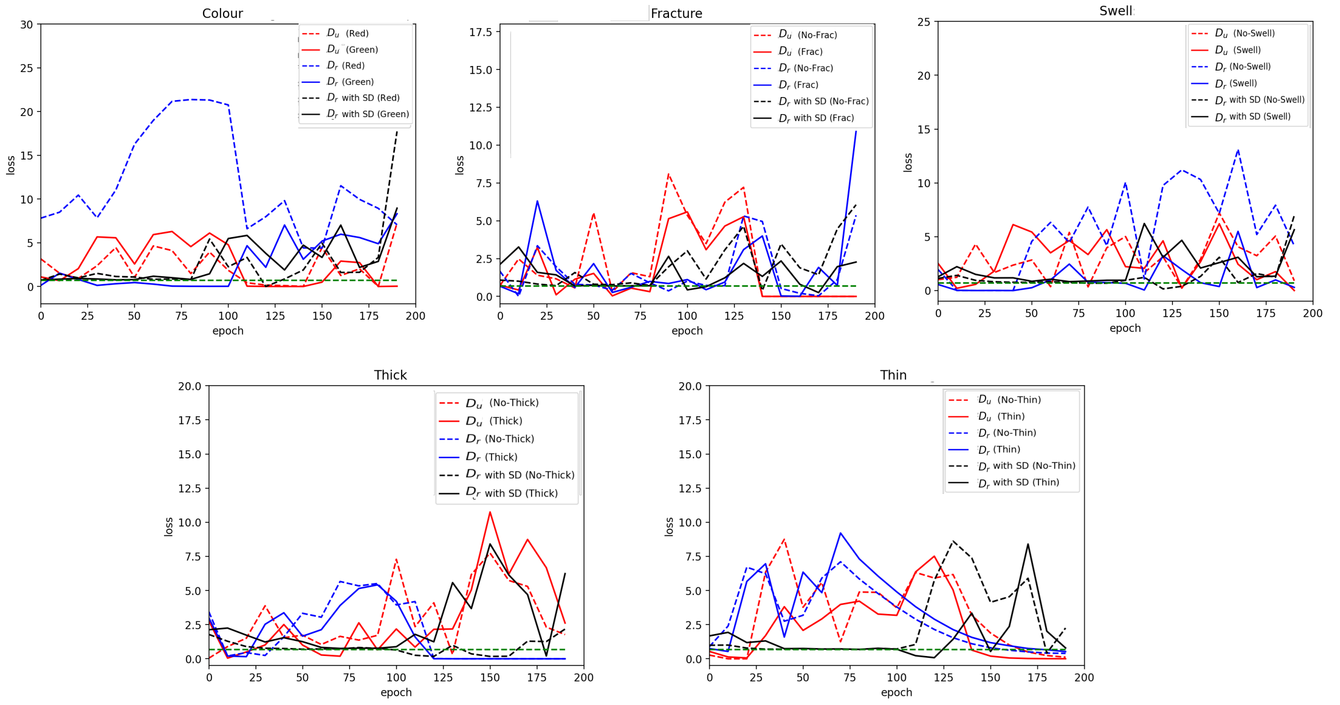


Figure S3: Discriminator loss with respect to the ”real label”, where RGF is introduced in digit “1”.

**Table S6.** GAN: z-scores (all images of digit “1” have RGF). Bold: similar proportions ($p > 0.05$), indicating RGF learning (With spectral decoupling)

| Digit | Colour | | | Fracture | | | Swell | | | Thick | | | Thin | | |
|---|---|---|---|---|---|---|---|---|---|---|---|---|---|---|---|
| | red | green | z | no | yes | z | no | yes | z | no | yes | z | no | yes | z |
| 0 | 232 | 0 | - | 97 | 21 | **1.36** | 88 | 17 | **0.89** | 114 | 0 | - | 78 | 15 | **0.82** |
| 1 | 73 | 87 | -5.49 | 22 | 95 | **1.44** | 65 | 69 | -5.68 | 47 | 48 | -4.97 | 20 | 66 | **0.16** |
| 2 | 25 | 0 | - | 44 | 36 | -2.34 | 46 | 60 | **-0.29** | 64 | 18 | -7.89 | 81 | 31 | -7.17 |
| 3 | 142 | 1 | -82.23 | 69 | 33 | -5.54 | 74 | 13 | -11.27 | 49 | 26 | -4.25 | 91 | 34 | -7.74 |
| 4 | 7 | 0 | - | 53 | 24 | -5.08 | 94 | 25 | -9.91 | 93 | 10 | -16.55 | 72 | 44 | -4.45 |
| 5 | 30 | 0 | - | 49 | 40 | -2.48 | 90 | 10 | -16.00 | 125 | 19 | -15.89 | 45 | 22 | -4.39 |
| 6 | 124 | 0 | - | 58 | 30 | -4.93 | 77 | 23 | -8.55 | 75 | 9 | -14.31 | 65 | 11 | -11.03 |
| 7 | 32 | 0 | - | 74 | 12 | -7.77 | 92 | 13 | -9.53 | 106 | 29 | -6.09 | 53 | 44 | **0.47** |
| 8 | 24 | 0 | - | 68 | 19 | -2.97 | 48 | 22 | **-0.64** | 70 | 18 | -3.38 | 88 | 6 | -11.35 |
| 9 | 223 | 0 | - | 120 | 36 | -6.50 | 54 | 20 | -3.48 | 73 | 7 | -11.47 | 109 | 25 | -7.83 |
| Total | 912 | 88 | -44.87 | 654 | 346 | -9.57 | 728 | 272 | -15.49 | 816 | 184 | -24.97 | 702 | 298 | -13.27 |

**Table S7.** GAN: z-scores (all images of digit “2” have RGF). Bold: similar proportions ($p > 0.05$), indicating RGF learning (Without spectral decoupling).

| Digit | Colour | | | Fracture | | | Swell | | | Thick | | | Thin | | |
|---|---|---|---|---|---|---|---|---|---|---|---|---|---|---|---|
| | red | green | z | no | yes | z | no | yes | z | no | yes | z | no | yes | z |
| 0 | 119 | 0 | - | 78 | 14 | **0.59** | 67 | 34 | -4.54 | 105 | 7 | -8.20 | 80 | 10 | -10.53 |
| 1 | 97 | 6 | -12.64 | 112 | 4 | -42.82 | 91 | 16 | -11.04 | 144 | 4 | -28.73 | 107 | 12 | -14.10 |
| 2 | 16 | 101 | 13.64 | 24 | 37 | **0.42** | 28 | 38 | **-0.40** | 24 | 54 | 9.99 | 23 | 76 | 7.25 |
| 3 | 84 | 2 | -25.03 | 47 | 57 | **-0.65** | 51 | 26 | -4.31 | 95 | 9 | -15.00 | 106 | 4 | -32.70 |
| 4 | 130 | 0 | - | 101 | 4 | -29.01 | 59 | 47 | -2.21 | 73 | 0 | - | 71 | 13 | -12.04 |
| 5 | 75 | 0 | - | 36 | 49 | **-0.07** | 43 | 27 | -4.89 | 84 | 1 | -43.46 | 84 | 16 | -16.09 |
| 6 | 122 | 1 | -49.63 | 126 | 14 | -19.33 | 86 | 61 | -6.03 | 94 | 9 | -27.05 | 78 | 11 | -17.38 |
| 7 | 87 | 1 | -35.28 | 118 | 8 | -16.87 | 75 | 32 | -4.31 | 114 | 11 | -12.31 | 91 | 11 | -17.33 |
| 8 | 86 | 2 | -45.78 | 72 | 21 | -2.86 | 82 | 42 | -8.50 | 35 | 21 | **-1.93** | 86 | 7 | -17.35 |
| 9 | 71 | 0 | - | 76 | 2 | -23.71 | 74 | 21 | -7.26 | 113 | 3 | -32.17 | 104 | 10 | -14.81 |
| Total | 887 | 113 | -37.66 | 790 | 210 | -21.74 | 656 | 344 | -15.71 | 881 | 119 | -34.28 | 830 | 170 | -35.36 |

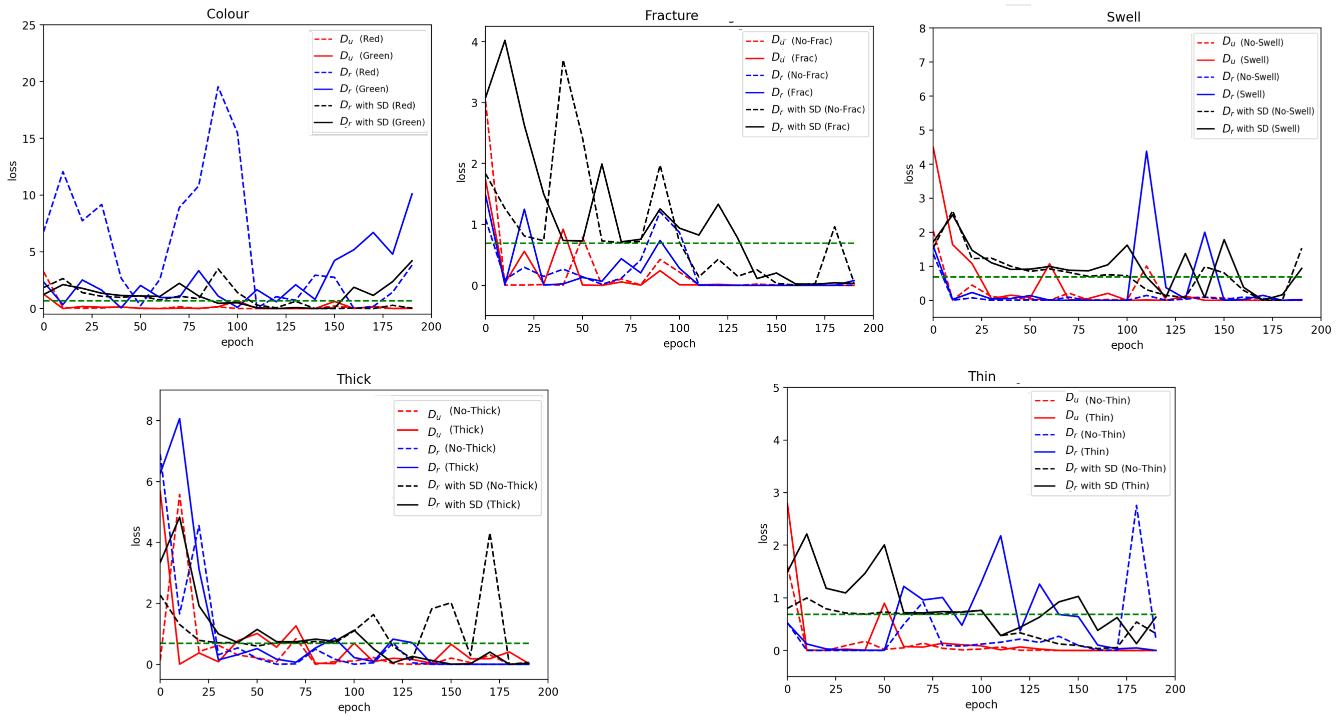


Figure S4: Discriminator loss with respect to the ”real label”, where RGF is introduced in digit “2”.

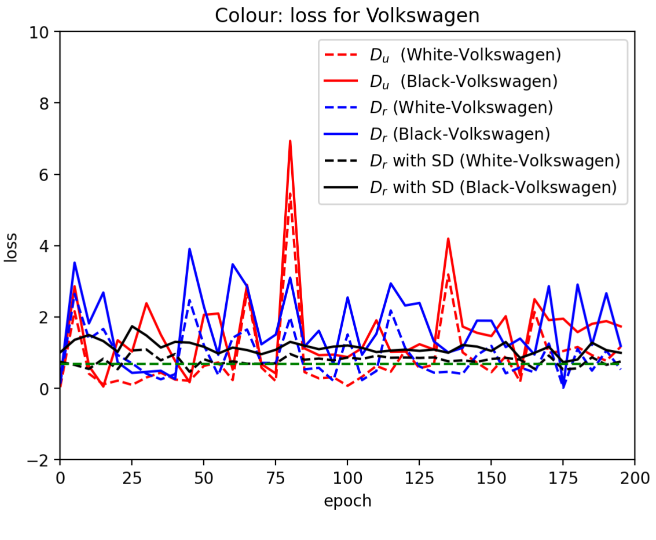


Figure S5: Discriminator loss with respect to the ”real label”, where RGF is introduced in Volkswagen.

We provided further visual insight into VAE results in Figure S17 and S18. These results indicate the memorization of RGF for skewed data.

Finally, we used the p-values corresponding to the z-scores in Tables S9 and S10 to deduce whether the VAE learn (L) or memorize (M) the RGFs. We observe that VAE memorizes less (learns in 21 cases) than GAN (learns in 9 cases).

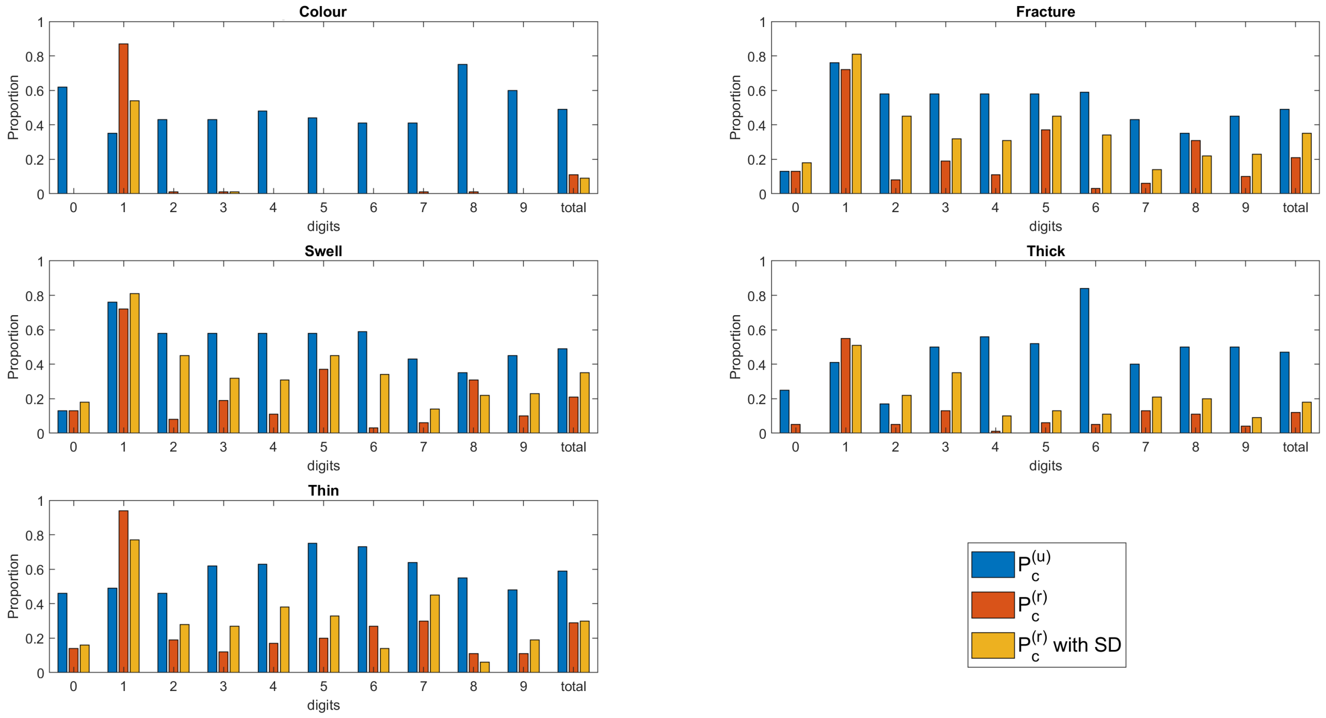


Figure S6: GAN: Generative factors proportions where the RGF is present in digit “1”.

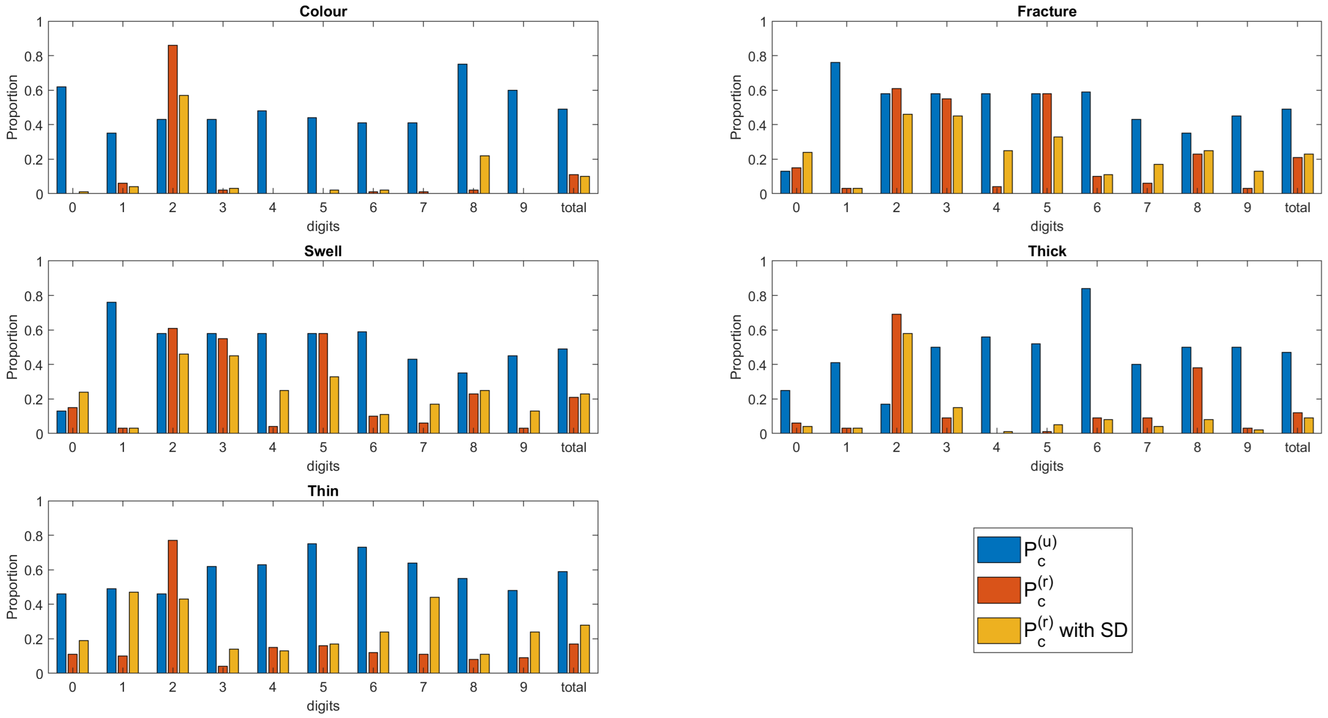


Figure S7: GAN: Generative factors proportions where the RGF is present in digit “2”.

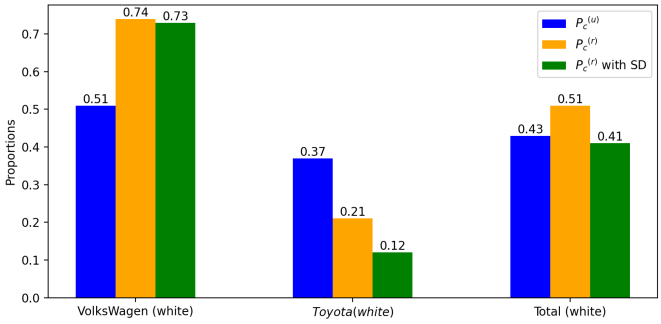


Figure S8: Generative factors proportions where the white colour is present in only Volkswagen and black is only in Toyota.

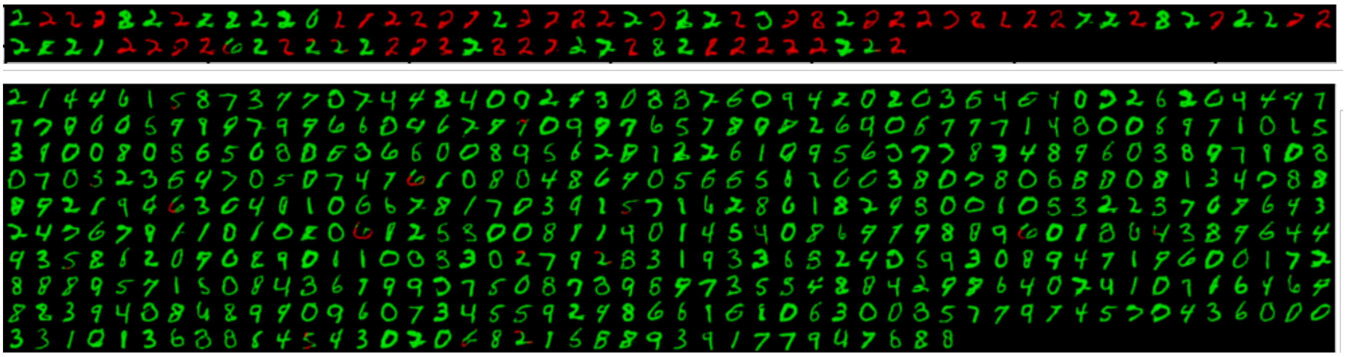

Figure S9: GAN generated images from training on the balanced dataset $D_u$. Top row: Images classified as digit “2” by the oracle classifier. Bottom row: Images classified as green by the colour classifier. Note that digit “2” appears in both colours (top row) and green images include various digits (bottom row)

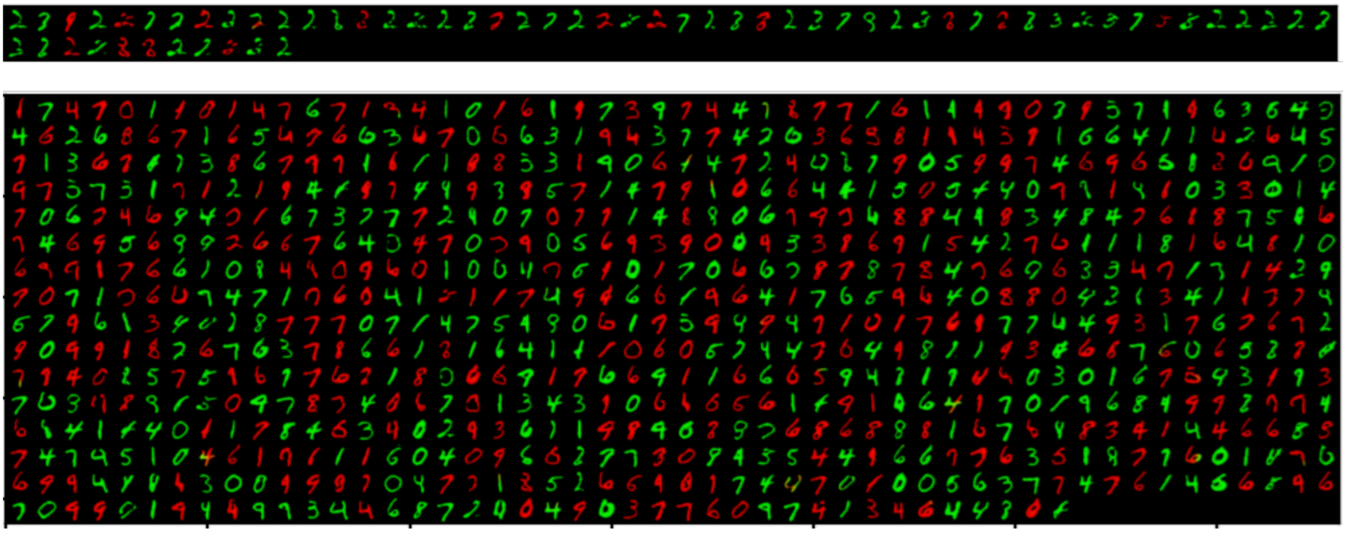

Figure S10: GAN generated images trained with fractured-RGF. Top row: Images classified as digit “2” by the oracle classifier. Bottom row: Images classified as non-fractured. Note that most “2” digits appear fractured (top row), while non-fractured images rarely contain “2” (bottom row)

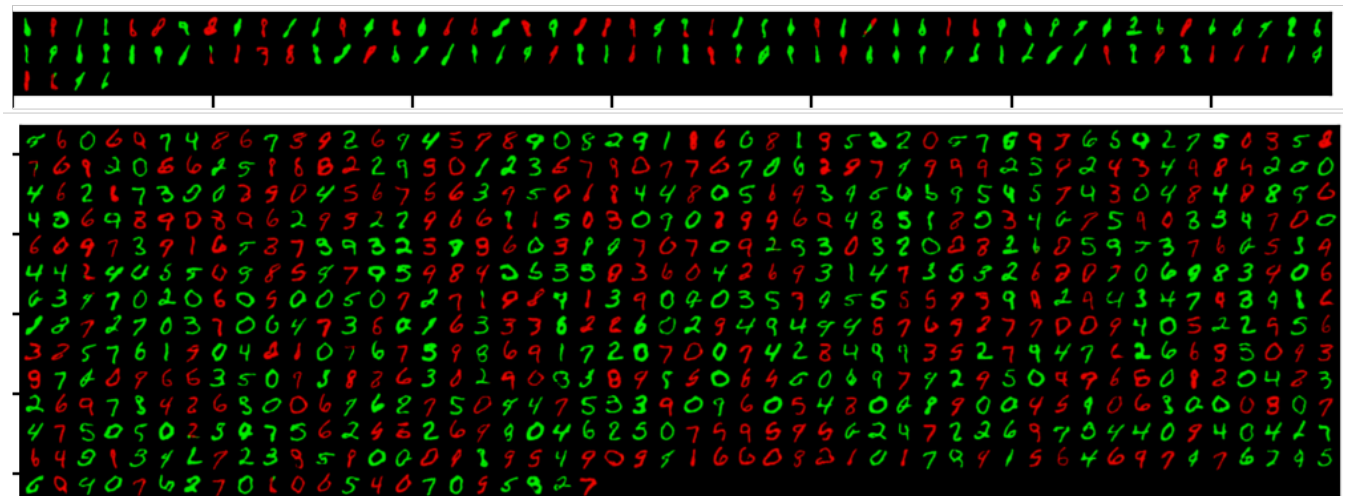

Figure S11: GAN generated images trained with swell-RGF. Top row: Images classified as digit “1” by the oracle classifier. Bottom row: Images classified as non-swelled. Note that most “1” digits appear swelled (top row), while non-swelled images rarely contain “1” (bottom row).

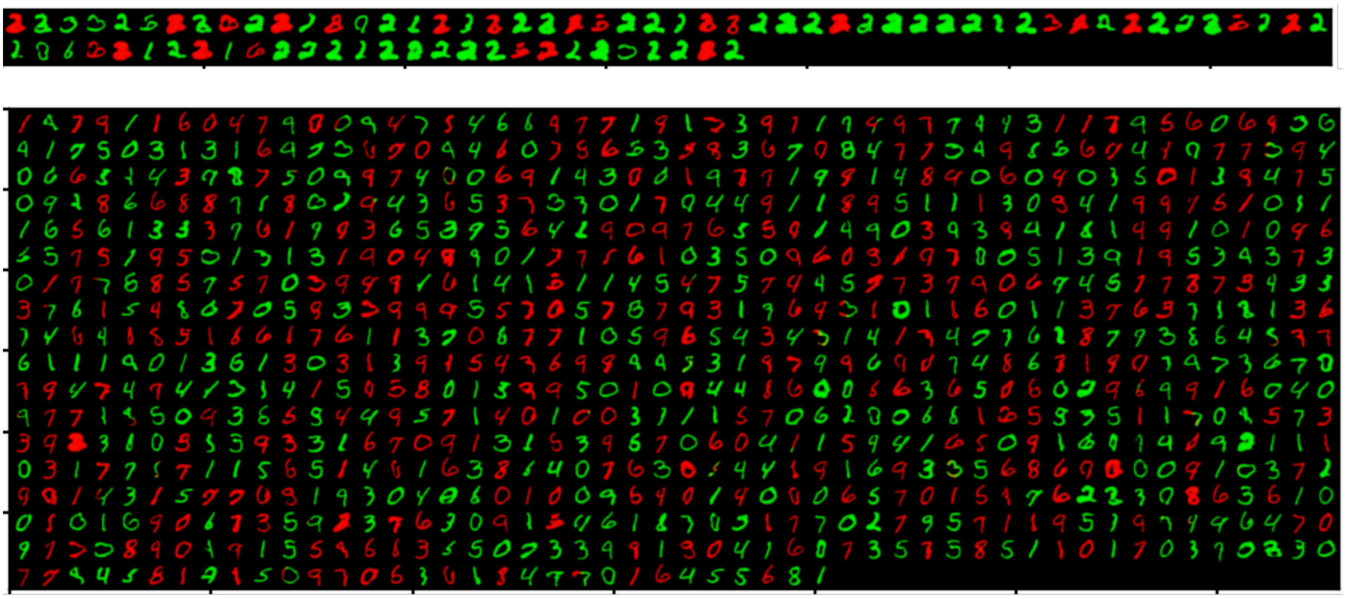

Figure S12: GAN generated images trained with thick-RGF. Top row: Images classified as digit “2” by the oracle classifier. Bottom row: Images classified as non-thick. Note that most “2” digits appear thick (top row), while non-thick images rarely contain “2” (bottom row).

## 4.3 Diffusion Model Results

For digit “2”, the results are shown in Table S12. Overall, the results demonstrate that diffusion models, like GANs and VAEs, exhibit a strong tendency to memorize rare generative factors rather than learn to apply them more broadly. This behaviour appears particularly pronounced for visually salient factors like colour.

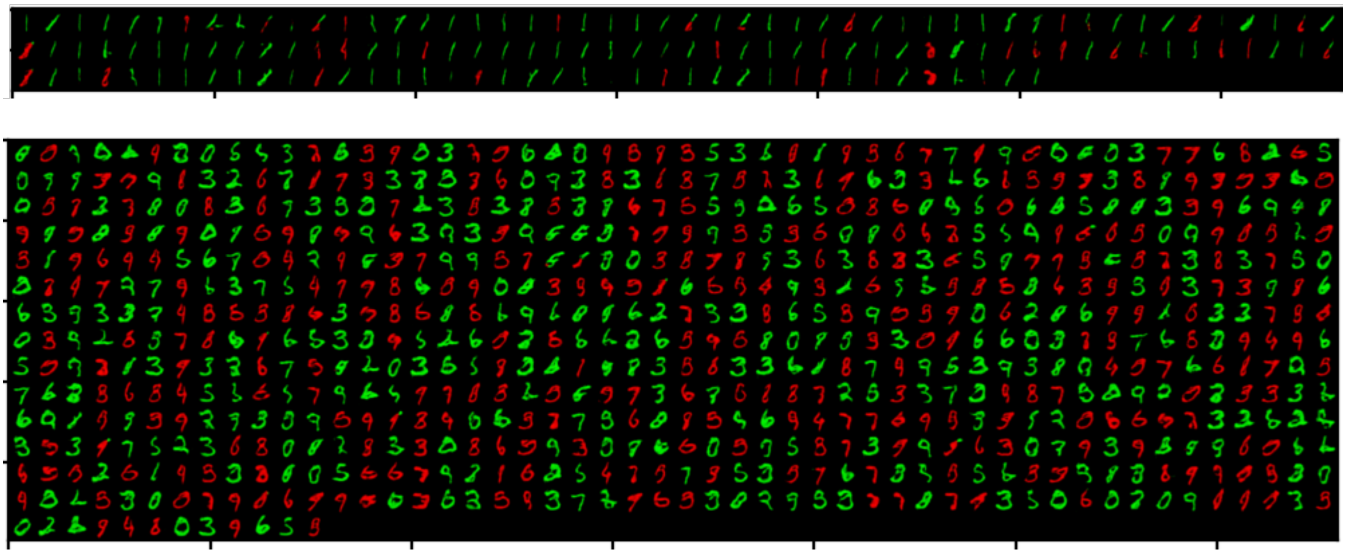

Figure S13: GAN generated images trained with thin-RGF. Top row: Images classified as digit “1” by the oracle classifier. Bottom row: Images classified as non-thin. Note that most “1” digits appear thin (top row), while non-thin images rarely contain “1” (bottom row).

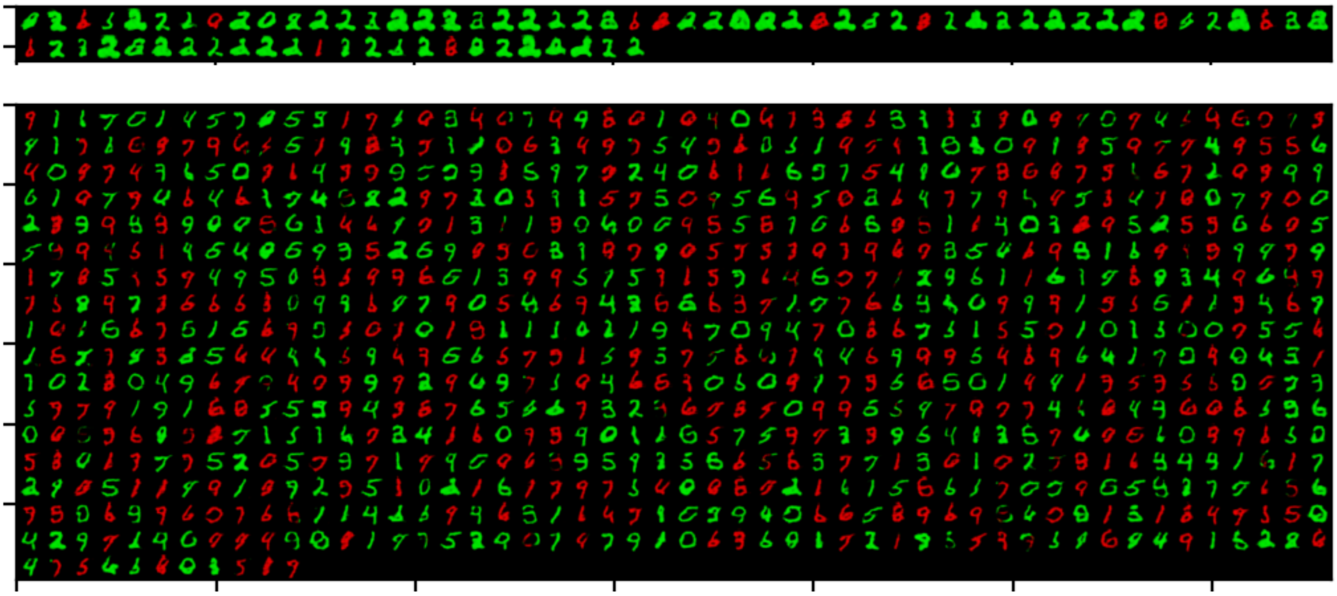

Figure S14: GAN generated images using **SD**, trained with thick-RGF. Top row: Images classified as digit “2” by the oracle classifier. Bottom row: Images classified as non-thick. Note that “2” digits appear in both thick and non-thick variants (top row), and non-thick images now include “2” digits (bottom row).

## 5 RELAX THE EXTREMITY

We have relaxed the rarity in the data, as shown in Table S13, S14, S15, S16, S17, S18, S19, S20 and S21. The results are summarized in Table S22 (93 cases learned out of 440), demonstrating an improvement in learning by relaxing data rarity. A z-proportion test ($p < 0.05$) confirmed this learning improvement compared to the extreme case, where only 43 cases were learned out of 440.

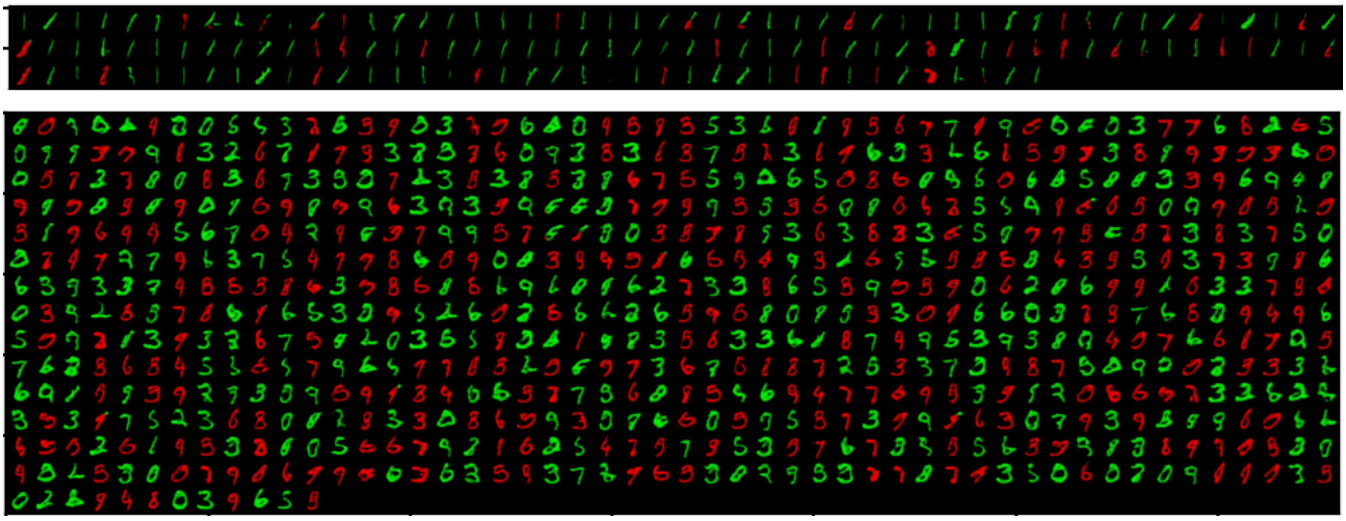

Figure S15: GAN generated images using **SD**, trained with thin-RGF. Top row: Images classified as digit "1" by the oracle classifier. Bottom row: Images classified as non-thin. Note that "1" digits appear in both thin and non-thin variants (top row), and non-thin images now include "1" digits (bottom row).

**Table S8.** GAN: z-scores (all images of digit "2" have RGF). Bold: similar proportions ($p > 0.05$), indicating RGF learning (With spectral decoupling)

| Digit | Colour | | | Fracture | | | Swell | | | Thick | | | Thin | | |
|---|---|---|---|---|---|---|---|---|---|---|---|---|---|---|---|
| | red | green | z | no | yes | z | no | yes | z | no | yes | z | no | yes | z |
| 0 | 126 | 1 | -78.05 | 81 | 26 | 2.73 | 80 | 30 | 3.36 | 101 | 4 | -4.92 | 107 | 25 | **1.74** |
| 1 | 197 | 8 | -22.99 | 87 | 3 | -38.40 | 109 | 34 | -14.67 | 103 | 3 | -45.43 | 52 | 47 | -5.68 |
| 2 | 51 | 68 | 3.12 | 50 | 42 | -2.38 | 37 | 39 | **-1.17** | 30 | 42 | **0.06** | 62 | 47 | -3.14 |
| 3 | 181 | 6 | -30.88 | 62 | 50 | -2.84 | 86 | 17 | -11.34 | 69 | 12 | -10.94 | 63 | 10 | -11.01 |
| 4 | 61 | 0 | - | 53 | 18 | -6.32 | 84 | 18 | -10.69 | 108 | 1 | -62.51 | 76 | 11 | -12.73 |
| 5 | 115 | 2 | -35.29 | 45 | 22 | -4.39 | 75 | 12 | -11.96 | 87 | 5 | -22.24 | 59 | 12 | -9.24 |
| 6 | 41 | 1 | -16.42 | 103 | 13 | -16.32 | 73 | 38 | -5.50 | 124 | 11 | -21.60 | 42 | 13 | -6.17 |
| 7 | 52 | 0 | - | 110 | 23 | -7.84 | 73 | 18 | -5.56 | 120 | 5 | -22.25 | 89 | 69 | **0.17** |
| 8 | 32 | 9 | -8.21 | 63 | 21 | -2.12 | 79 | 18 | -4.17 | 72 | 6 | -9.05 | 71 | 9 | -6.72 |
| 9 | 49 | 0 | - | 112 | 16 | -11.12 | 72 | 8 | -10.43 | 95 | 2 | -29.76 | 104 | 32 | -5.90 |
| Total | 905 | 95 | -42.60 | 766 | 234 | -19.12 | 768 | 232 | -19.33 | 909 | 91 | -43.87 | 725 | 275 | -15.23 |

**Table S9.** VAE: z-scores (all images of digit "1" have RGF). Bold: similar proportions ($p > 0.05$), indicating RGF learning.

| Digit | Colour | | | Fracture | | | Swell | | | Thick | | | Thin | | |
|---|---|---|---|---|---|---|---|---|---|---|---|---|---|---|---|
| | red | green | z | no | yes | z | no | yes | z | no | yes | z | no | yes | z |
| 0 | 105 | 0 | - | 75 | 24 | **-1.80** | 82 | 6 | -5.28 | 59 | 11 | -4.66 | 65 | 51 | -3.70 |
| 1 | 57 | 17 | -6.14 | 39 | 38 | 3.92 | 41 | 60 | **-0.94** | 48 | 45 | 2.39 | 3 | 47 | 7.15 |
| 2 | 94 | 0 | - | 78 | 27 | **-1.71** | 73 | 8 | -8.48 | 84 | 13 | -7.11 | 75 | 64 | -2.36 |
| 3 | 103 | 3 | -24.94 | 67 | 24 | -2.30 | 80 | 15 | -2.19 | 73 | 6 | -12.21 | 60 | 27 | -3.62 |
| 4 | 121 | 0 | - | 70 | 51 | **0.03** | 81 | 17 | -7.23 | 110 | 27 | -5.97 | 50 | 59 | **-1.23** |
| 5 | 87 | 0 | - | 52 | 56 | **0.59** | 74 | 4 | -3.55 | 119 | 4 | -22.98 | 51 | 54 | -3.60 |
| 6 | 100 | 0 | - | 97 | 37 | **-1.65** | 99 | 29 | -3.07 | 95 | 10 | -12.03 | 65 | 45 | -5.57 |
| 7 | 105 | 0 | - | 73 | 25 | **-0.79** | 98 | 19 | -10.78 | 79 | 42 | -2.38 | 57 | 49 | **-0.78** |
| 8 | 94 | 19 | -10.29 | 86 | 8 | -2.25 | 100 | 33 | -5.66 | 56 | 41 | **-1.34** | 62 | 23 | -5.59 |
| 9 | 95 | 0 | - | 65 | 8 | -5.48 | 68 | 13 | -8.57 | 52 | 26 | **-1.62** | 46 | 47 | **-1.25** |
| Total | 961 | 39 | -75.30 | 702 | 298 | -2.21 | 796 | 204 | -14.60 | 775 | 225 | -14.01 | 534 | 466 | -7.86 |

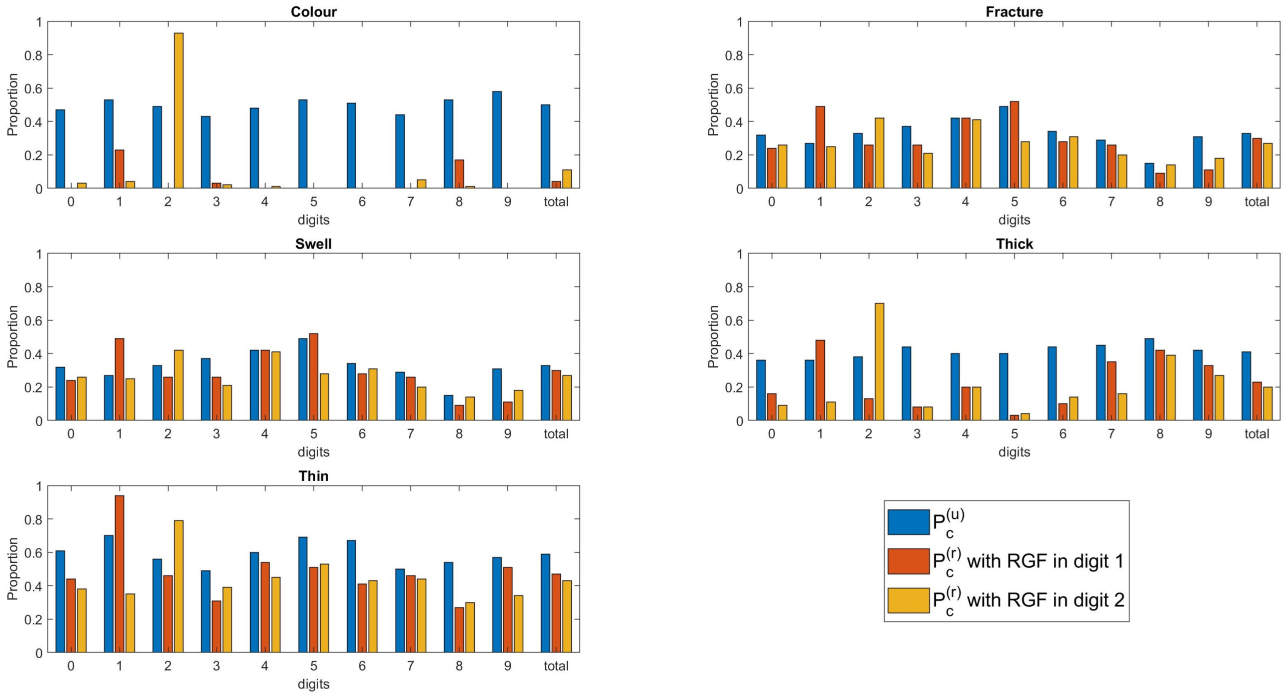


Figure S16: VAE: Generative factors proportions

**Table S10.** VAE: z-scores (all images of digit “2” have RGF). Bold: similar proportions ($p > 0.05$), indicating RGF learning.

| Digit | Colour | | | Fracture | | | Swell | | | Thick | | | Thin | | |
|---|---|---|---|---|---|---|---|---|---|---|---|---|---|---|---|
| | red | green | z | no | yes | z | no | yes | z | no | yes | z | no | yes | z |
| 0 | 64 | 2 | -20.84 | 54 | 19 | **-1.16** | 70 | 12 | **-1.63** | 106 | 11 | -9.86 | 46 | 28 | -4.11 |
| 1 | 90 | 4 | -23.41 | 51 | 17 | **-0.38** | 63 | 19 | -8.76 | 68 | 8 | -7.24 | 55 | 29 | -6.84 |
| 2 | 7 | 93 | 17.24 | 69 | 49 | **1.88** | 56 | 63 | 3.27 | 23 | 54 | 6.16 | 18 | 66 | 5.04 |
| 3 | 90 | 2 | -26.85 | 81 | 21 | -4.10 | 77 | 9 | -4.10 | 94 | 8 | -13.58 | 71 | 45 | -2.26 |
| 4 | 92 | 1 | -43.88 | 74 | 51 | **-0.27** | 84 | 22 | -6.16 | 84 | 21 | -5.12 | 77 | 62 | -3.65 |
| 5 | 102 | 0 | - | 62 | 24 | -4.36 | 121 | 12 | -2.00 | 132 | 5 | -22.69 | 38 | 43 | -2.87 |
| 6 | 136 | 0 | - | 81 | 36 | **-0.76** | 86 | 29 | -2.17 | 106 | 17 | -9.70 | 69 | 52 | -5.34 |
| 7 | 94 | 5 | -17.70 | 80 | 20 | -2.25 | 106 | 10 | -17.03 | 81 | 15 | -7.93 | 59 | 46 | **-1.28** |
| 8 | 105 | 1 | -55.44 | 98 | 16 | **-0.30** | 72 | 13 | -7.87 | 57 | 37 | **-1.91** | 62 | 26 | -5.03 |
| 9 | 112 | 0 | - | 80 | 17 | -3.49 | 63 | 13 | -7.85 | 53 | 20 | -2.80 | 71 | 37 | -4.98 |
| Total | 892 | 108 | -39.94 | 730 | 270 | -4.27 | 798 | 202 | -14.81 | 804 | 196 | -17.05 | 566 | 434 | -9.95 |

**Table S11.** DM: z-scores (all images of digit “1” have RGF). Bold: similar proportions ($p > 0.05$), indicating RGF learning.

| Digit | Colour | | | Fracture | | | Swell | | | Thick | | | Thin | | |
|---|---|---|---|---|---|---|---|---|---|---|---|---|---|---|---|
| | red | green | z | no | yes | z | no | yes | z | no | yes | z | no | yes | z |
| 0 | 126 | 0 | - | 103 | 2 | -28.56 | 105 | 14 | -6.51 | 110 | 5 | -9.28 | 116 | 4 | -40.68 |
| 1 | 4 | 99 | 14.77 | 4 | 116 | 32.75 | 18 | 90 | 9.57 | 8 | 128 | 26.33 | 9 | 104 | 14.54 |
| 2 | 98 | 0 | - | 80 | 36 | -4.42 | 92 | 20 | -9.43 | 95 | 9 | -12.1 | 88 | 15 | -16.81 |
| 3 | 82 | 0 | - | 87 | 12 | -14.9 | 62 | 21 | -6.85 | 57 | 3 | -14.93 | 77 | 1 | -50.81 |
| 4 | 75 | 0 | - | 68 | 1 | -37.92 | 72 | 15 | -7.6 | 80 | 8 | -8.45 | 93 | 8 | -15.66 |
| 5 | 113 | 0 | - | 70 | 9 | -11.92 | 77 | 10 | -9.21 | 81 | 1 | -20.45 | 58 | 14 | -12.13 |
| 6 | 119 | 0 | - | 94 | 4 | -16.97 | 72 | 22 | -5.63 | 85 | 6 | -14.76 | 109 | 1 | -66.4 |
| 7 | 103 | 0 | - | 118 | 8 | -14.11 | 97 | 23 | -13.31 | 100 | 4 | -22.88 | 116 | 15 | -19.25 |
| 8 | 129 | 0 | - | 119 | 4 | -14.22 | 111 | 34 | -7.26 | 145 | 12 | -23.75 | 112 | 8 | -13.32 |
| 9 | 52 | 0 | - | 63 | 2 | -14.44 | 36 | 9 | -7.04 | 53 | 10 | -15.23 | 40 | 12 | -6.49 |
| Total | 901 | 99 | -42.67 | 806 | 194 | -18.87 | 742 | 258 | -17.49 | 814 | 186 | -20.64 | 818 | 182 | -35.08 |

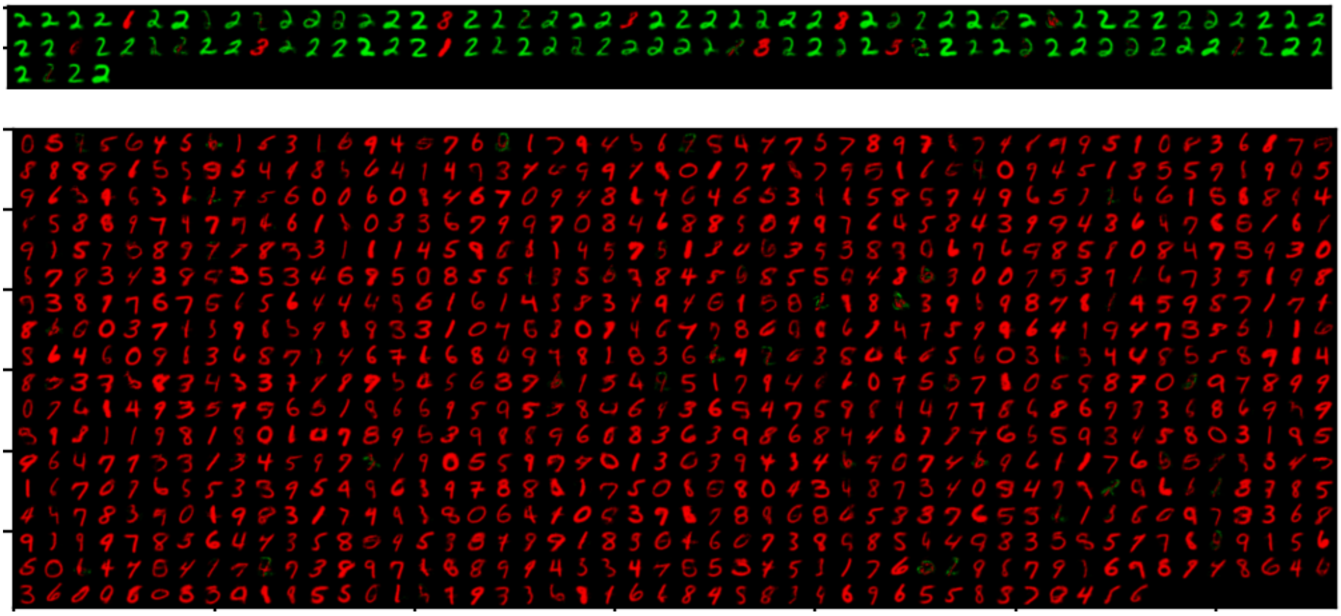

Figure S17: VAE generated images trained with colored-RGF. Top row: Images classified as digit "2" by the oracle classifier. Bottom row: Images classified as red. Note that most "2" digits appear green (top row), while red images rarely contain "2" (bottom row).

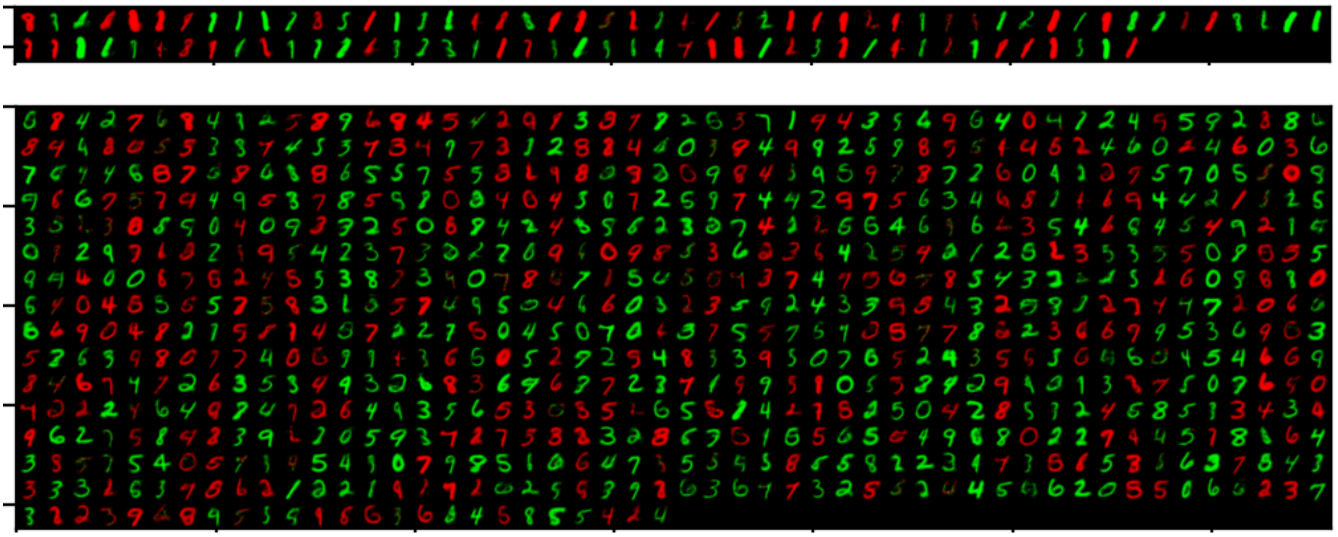

Figure S18: VAE generated images trained with thick-RGF. Top row: Images classified as digit "1" by the oracle classifier. Bottom row: Images classified as non-thick. Note that most "1" digits appear thick (top row), while non-thick images rarely contain "1" (bottom row).

Figure S20: Comparison of discriminator loss when applying different $\lambda$ values. (Top) Digit "1" with RGF. (Bottom) Volkswagen Car with RGF.

## 6 SEEDS

We have tried two different seeds and summarised the findings in Table S39 (53 learned cases out of 440) and S40 (51 out of 440). No significant difference ($p < 0.05$) is observed for the total number of cases of RGFs learning using proportion test.

**Table S12.** DM: RGF in digit "2". Bold: similar proportions ($p > 0.05$), indicating RGF learning.

| Digit | Colour | | | Fracture | | | Swell | | | Thick | | | Thin | | |
|---|---|---|---|---|---|---|---|---|---|---|---|---|---|---|---|
| | red | green | z | no | yes | z | no | yes | z | no | yes | z | no | yes | z |
| 0 | 118 | 0 | - | 117 | 4 | -22.58 | 115 | 2 | -24.44 | 101 | 1 | -21.55 | 120 | 4 | -42.08 |
| 1 | 131 | 1 | -89.1 | 126 | 4 | -26.36 | 109 | 12 | -14.38 | 117 | 8 | -15.81 | 107 | 8 | -20.25 |
| 2 | 2 | 82 | 42.09 | 46 | 65 | **1.83** | 14 | 74 | 8.23 | 24 | 107 | 11.74 | 4 | 127 | 15.93 |
| 3 | 47 | 0 | - | 58 | 21 | -6.92 | 71 | 28 | -6.57 | 79 | 1 | -36.83 | 68 | 6 | -18.24 |
| 4 | 83 | 0 | - | 82 | 18 | -9.89 | 90 | 24 | -7.06 | 94 | 10 | -8.78 | 62 | 8 | -10.14 |
| 5 | 112 | 0 | - | 62 | 35 | -3.67 | 71 | 2 | -21.07 | 60 | 0 | - | 66 | 17 | -12.53 |
| 6 | 109 | 0 | - | 91 | 8 | -10.92 | 65 | 14 | -7.05 | 99 | 13 | -11.03 | 87 | 3 | -30.48 |
| 7 | 122 | 1 | -70.75 | 81 | 10 | -7.93 | 80 | 25 | -10.39 | 92 | 9 | -13.44 | 107 | 11 | -20.8 |
| 8 | 145 | 1 | -69.9 | 118 | 11 | -7.11 | 116 | 34 | -7.7 | 136 | 12 | -22.24 | 119 | 5 | -18.66 |
| 9 | 46 | 0 | - | 41 | 2 | -9.14 | 44 | 10 | -8.23 | 30 | 7 | -10.42 | 60 | 11 | -10.6 |
| Total | 915 | 85 | -47.28 | 822 | 178 | -20.83 | 775 | 225 | -20.83 | 832 | 168 | -23.01 | 800 | 200 | -32.41 |

**Table S13.** VAE results with RGF in digit "1", rarity relaxed to 20%. Bold: similar proportions ($p > 0.05$), indicating RGF learning.

| **Digits** | **colour** | | | **frac** | | | **swel** | | | **Thick** | | | **Thin** | | |
|---|---|---|---|---|---|---|---|---|---|---|---|---|---|---|---|
| | **Red** | **Green** | **z** | **no** | **yes** | **z** | **no** | **yes** | **z** | **no** | **yes** | **z** | **no** | **yes** | **z** |
| 0 | 76 | 18 | -8.34 | 93 | 32 | **-1.64** | 51 | 52 | 5.99 | 53 | 42 | **1.61** | 76 | 35 | -6.68 |
| 1 | 50 | 31 | **-1.62** | 65 | 34 | **1.54** | 33 | 65 | **0.49** | 44 | 46 | 2.87 | 28 | 59 | **-0.44** |
| 2 | 82 | 23 | -7.21 | 70 | 46 | **1.47** | 60 | 47 | **1.23** | 60 | 32 | **-0.65** | 81 | 43 | -4.99 |
| 3 | 101 | 20 | -11.99 | 52 | 37 | **0.88** | 54 | 45 | 4.29 | 58 | 24 | -2.93 | 69 | 32 | -3.74 |
| 4 | 68 | 18 | -7.08 | 45 | 48 | **1.86** | 39 | 31 | **-0.12** | 79 | 34 | -2.3 | 54 | 40 | -3.42 |
| 5 | 74 | 16 | -7.25 | 41 | 45 | **0.62** | 56 | 35 | 4.8 | 74 | 30 | -2.51 | 54 | 40 | -5.19 |
| 6 | 73 | 18 | -7 | 57 | 20 | **-1.61** | 33 | 36 | 3.02 | 50 | 34 | **-0.66** | 50 | 28 | -5.73 |
| 7 | 83 | 28 | -7.47 | 59 | 41 | 2.44 | 72 | 46 | -3.12 | 69 | 27 | -3.68 | 54 | 44 | **-1.02** |
| 8 | 89 | 19 | -8.03 | 84 | 40 | 4.11 | 68 | 58 | **0.01** | 83 | 55 | -2.19 | 69 | 22 | -6.64 |
| 9 | 86 | 27 | -4.51 | 71 | 20 | -2.08 | 81 | 38 | -4.46 | 69 | 37 | **-1.53** | 79 | 43 | -5.03 |
| T | 782 | 218 | -21.6 | 637 | 363 | 2.17 | 547 | 453 | 4 | 639 | 361 | -3.23 | 614 | 386 | -13.25 |

**Table S14.** VAE results with RGF in digit "2", rarity relaxed to 20%. Bold: similar proportions ($p > 0.05$), indicating RGF learning.

| **Digits** | **colour** | | | **frac** | | | **swel** | | | **Thick** | | | **Thin** | | |
|---|---|---|---|---|---|---|---|---|---|---|---|---|---|---|---|
| | **Red** | **Green** | **z** | **no** | **yes** | **z** | **no** | **yes** | **z** | **no** | **yes** | **z** | **no** | **yes** | **z** |
| 0 | 74 | 30 | -5.44 | 68 | 16 | -3.02 | 58 | 31 | 2.74 | 50 | 37 | **1.23** | 46 | 52 | -1.57 |
| 1 | 81 | 24 | -5.89 | 66 | 15 | **-1.97** | 73 | 33 | -7.31 | 87 | 29 | -2.74 | 68 | 43 | -6.76 |
| 2 | 44 | 33 | **-1.44** | 84 | 48 | **0.8** | 48 | 45 | **2** | 57 | 40 | **0.65** | 38 | 55 | **0.62** |
| 3 | 67 | 27 | -6.06 | 60 | 28 | **-1.04** | 61 | 32 | 2.11 | 71 | 20 | -5.07 | 52 | 46 | **-0.41** |
| 4 | 61 | 20 | -5.7 | 54 | 46 | **0.8** | 52 | 34 | **-1.04** | 70 | 30 | -2.18 | 51 | 52 | **-1.93** |
| 5 | 82 | 27 | -5.38 | 44 | 31 | **-1.35** | 73 | 31 | 3.52 | 65 | 22 | -3.16 | 44 | 48 | -3.23 |
| 6 | 74 | 22 | -6.08 | 72 | 24 | -2.04 | 54 | 37 | **1.29** | 64 | 25 | -3.34 | 35 | 33 | -3.05 |
| 7 | 86 | 23 | -8.93 | 73 | 24 | **-0.97** | 73 | 33 | -4.86 | 70 | 26 | -3.95 | 61 | 62 | **0.09** |
| 8 | 82 | 26 | -5.57 | 114 | 36 | 2.58 | 81 | 39 | -3.16 | 82 | 55 | -2.11 | 61 | 37 | -3.32 |
| 9 | 92 | 25 | -5.44 | 62 | 35 | **1.04** | 80 | 32 | -5.25 | 63 | 37 | **-1.04** | 66 | 50 | -3.02 |
| T | 743 | 257 | -17.59 | 697 | 303 | **-1.86** | 653 | 347 | -2.86 | 679 | 321 | -6.03 | 522 | 478 | -7.09 |

**Table S15.** GAN results with RGF in digit "1", rarity relaxed to 20%. Bold: similar proportions ($p > 0.05$), indicating RGF learning (Without spectral decoupling).

| Digit | Colour | | | Fracture | | | Swell | | | Thick | | | Thin | | |
|---|---|---|---|---|---|---|---|---|---|---|---|---|---|---|---|
| | red | green | z | no | yes | z | no | yes | z | no | yes | z | no | yes | z |
| 0 | 78 | 18 | -4.83 | 58 | 28 | 3.87 | 85 | 27 | -7.64 | 71 | 54 | 4.11 | 53 | 80 | 3.33 |
| 1 | 33 | 88 | **1.91** | 71 | 25 | -11.15 | 38 | 36 | **-0.75** | 47 | 25 | **-1.12** | 42 | 48 | **0.82** |
| 2 | 73 | 25 | -7.15 | 60 | 32 | -4.68 | 54 | 38 | -3.64 | 62 | 36 | 4.05 | 47 | 129 | 8.18 |
| 3 | 68 | 27 | -6.18 | 80 | 32 | -6.89 | 77 | 30 | -6.67 | 59 | 31 | -3.11 | 63 | 23 | -7.39 |
| 4 | 79 | 12 | -10.94 | 70 | 19 | -8.44 | 58 | 31 | -3.99 | 55 | 18 | -6.21 | 4 | 2 | **-1.54** |
| 5 | 84 | 25 | -8.21 | 68 | 20 | -7.9 | 66 | 31 | -7.4 | 67 | 25 | -5.35 | 80 | 20 | -13.75 |
| 6 | 82 | 22 | -9.45 | 27 | 75 | 3.33 | 39 | 73 | **-0.18** | 37 | 53 | -4.84 | 46 | 4 | -16.94 |
| 7 | 69 | 23 | -7.53 | 36 | 85 | 6.56 | 31 | 68 | 4.22 | 46 | 56 | 3.02 | 161 | 65 | -11.7 |
| 8 | 71 | 12 | -2.73 | 38 | 51 | 4.25 | 30 | 65 | **-0.33** | 55 | 87 | 2.76 | 10 | 100 | 13.1 |
| 9 | 84 | 27 | -3.85 | 53 | 72 | 2.85 | 57 | 66 | **0.15** | 53 | 63 | **0.93** | 9 | 14 | **1.26** |
| T | 721 | 279 | -16.29 | 561 | 439 | -3.25 | 535 | 465 | -7.29 | 552 | 448 | **-1.4** | 515 | 485 | -6.64 |

**Table S16.** GAN results with RGF in digit "1", rarity relaxed to 20%. Bold: similar proportions ($p > 0.05$), indicating RGF learning (With spectral decoupling).

| Digit | Colour | | | Fracture | | | Swell | | | Thick | | | Thin | | |
|---|---|---|---|---|---|---|---|---|---|---|---|---|---|---|---|
| | red | green | z | no | yes | z | no | yes | z | no | yes | z | no | yes | z |
| 0 | 86 | 19 | -5.3 | 77 | 43 | 5.22 | 55 | 43 | -2.22 | 99 | 37 | **0.58** | 91 | 38 | -4.12 |
| 1 | 26 | 71 | **1.82** | 73 | 30 | -10.47 | 47 | 34 | -2.01 | 61 | 24 | -2.61 | 74 | 41 | -2.99 |
| 2 | 70 | 21 | -7.68 | 58 | 41 | -3.35 | 70 | 29 | -6.71 | 73 | 31 | 2.86 | 68 | 32 | -3 |
| 3 | 68 | 32 | -5.36 | 65 | 36 | -4.69 | 74 | 38 | -5.16 | 62 | 33 | -3.12 | 72 | 32 | -6.9 |
| 4 | 69 | 11 | -9.93 | 71 | 33 | -5.76 | 51 | 37 | -2.46 | 58 | 30 | -4.34 | 31 | 36 | **-1.52** |
| 5 | 73 | 19 | -8.38 | 59 | 29 | -5 | 59 | 43 | -5.08 | 51 | 25 | -3.55 | 60 | 44 | -6.75 |
| 6 | 92 | 21 | -11.05 | 40 | 64 | **0.53** | 43 | 69 | **-0.96** | 42 | 72 | -4.61 | 38 | 51 | -2.99 |
| 7 | 74 | 27 | -7.33 | 42 | 62 | 3.45 | 36 | 71 | 3.8 | 27 | 51 | 4.71 | 39 | 71 | **0.12** |
| 8 | 55 | 18 | **-0.07** | 35 | 59 | 5.57 | 51 | 58 | -3.51 | 30 | 75 | 4.86 | 28 | 41 | **0.75** |
| 9 | 111 | 37 | -4.21 | 36 | 47 | 2.14 | 33 | 59 | 2.23 | 49 | 70 | **1.96** | 55 | 58 | **0.71** |
| T | 724 | 276 | -16.55 | 556 | 444 | -2.93 | 519 | 481 | -6.27 | 552 | 448 | **-1.4** | 556 | 444 | -9.29 |

**Table S17.** GAN results with RGF in digit "2", rarity relaxed to 20%. Bold: similar proportions ($p > 0.05$), indicating RGF learning (Without spectral decoupling).

| Digit | Colour | | | Fracture | | | Swell | | | Thick | | | Thin | | |
|---|---|---|---|---|---|---|---|---|---|---|---|---|---|---|---|
| | red | green | z | no | yes | z | no | yes | z | no | yes | z | no | yes | z |
| 0 | 89 | 45 | **-1.08** | 52 | 40 | 5.9 | 53 | 33 | -3.17 | 66 | 36 | 2.18 | 39 | 37 | **0.47** |
| 1 | 96 | 27 | -11.53 | 55 | 43 | -6.41 | 66 | 36 | -3.74 | 72 | 43 | **-0.8** | 90 | 55 | -2.75 |
| 2 | 11 | 51 | 5.21 | 58 | 38 | -3.69 | 51 | 34 | -3.76 | 42 | 30 | 4.25 | 48 | 24 | -2.28 |
| 3 | 79 | 4 | -22.2 | 62 | 43 | -3.55 | 86 | 38 | -6.37 | 75 | 34 | -4.24 | 62 | 30 | -6.01 |
| 4 | 96 | 9 | -15.9 | 57 | 27 | -5.07 | 48 | 36 | -2.25 | 43 | 16 | -4.99 | 56 | 40 | -4.24 |
| 5 | 68 | 1 | -37.92 | 60 | 30 | -4.96 | 59 | 28 | -6.95 | 61 | 36 | -3.03 | 52 | 30 | -7.22 |
| 6 | 102 | 24 | -11.42 | 57 | 69 | **-0.96** | 45 | 73 | **-0.92** | 43 | 46 | -6.1 | 55 | 67 | -4.01 |
| 7 | 95 | 2 | -39.46 | 41 | 67 | 4.08 | 39 | 64 | 2.75 | 54 | 50 | **1.65** | 46 | 71 | **-0.73** |
| 8 | 78 | 11 | -3.62 | 32 | 52 | 5.08 | 21 | 50 | **0.08** | 35 | 90 | 5.48 | 40 | 55 | **0.57** |
| 9 | 55 | 57 | 2.31 | 46 | 71 | 3.47 | 52 | 88 | 2.41 | 47 | 81 | 3.12 | 42 | 61 | 2.32 |
| T | 769 | 231 | -20.93 | 520 | 480 | **-0.63** | 520 | 480 | -6.33 | 538 | 462 | **-0.51** | 530 | 470 | -7.6 |

**Table S18.** GAN results with RGF in digit "2", rarity relaxed to 20%. Bold: similar proportions ($p > 0.05$), indicating RGF learning (With spectral decoupling).

| Digit | Colour | | | Fracture | | | Swell | | | Thick | | | Thin | | |
|---|---|---|---|---|---|---|---|---|---|---|---|---|---|---|---|
| | red | green | z | no | yes | z | no | yes | z | no | yes | z | no | yes | z |
| 0 | 71 | 23 | -3.05 | 75 | 32 | 3.82 | 76 | 39 | -4.78 | 85 | 53 | 3.24 | 57 | 47 | **-0.17** |
| 1 | 94 | 25 | -11.78 | 63 | 41 | -7.63 | 74 | 32 | -5.12 | 62 | 62 | **2** | 76 | 46 | -2.57 |
| 2 | 18 | 66 | 4.82 | 43 | 33 | -2.56 | 54 | 44 | -3.01 | 54 | 33 | 4.02 | 49 | 38 | **-0.44** |
| 3 | 84 | 20 | -9.77 | 64 | 33 | -4.98 | 59 | 33 | -4.23 | 82 | 37 | -4.46 | 71 | 55 | -4.15 |
| 4 | 85 | 13 | -11.3 | 58 | 33 | -4.31 | 55 | 25 | -4.58 | 42 | 16 | -4.84 | 57 | 36 | -4.81 |
| 5 | 92 | 13 | -13.57 | 69 | 27 | -6.51 | 57 | 28 | -6.68 | 30 | 33 | **0.06** | 49 | 33 | -6.42 |
| 6 | 91 | 20 | -11.23 | 57 | 69 | **-0.96** | 45 | 73 | **-0.92** | 24 | 78 | **-1.79** | 41 | 67 | -2.35 |
| 7 | 68 | 32 | -5.79 | 39 | 60 | 3.59 | 38 | 63 | 2.77 | 21 | 85 | 10.38 | 47 | 70 | **-0.92** |
| 8 | 63 | 26 | **0.87** | 31 | 63 | 6.6 | 34 | 55 | **-1.59** | 20 | 53 | 4.33 | 20 | 41 | 2.03 |
| 9 | 73 | 23 | -3.68 | 38 | 72 | 4.51 | 39 | 77 | 3.05 | 42 | 88 | 4.31 | 46 | 54 | **1.2** |
| T | 739 | 261 | -17.93 | 537 | 463 | **-1.71** | 531 | 469 | -7.03 | 462 | 538 | 4.31 | 513 | 487 | -6.52 |

**Table S19.** DM results with RGF in digit "1", rarity relaxed to 20%. Bold: similar proportions ($p > 0.05$), indicating RGF learning.

| Digit | **colour** | | | **frac** | | | **swel** | | | **Thick** | | | **Thin** | | |
|---|---|---|---|---|---|---|---|---|---|---|---|---|---|---|---|
| | **red** | **green** | **z** | **no** | **yes** | **z** | **no** | **yes** | **z** | **no** | **yes** | **z** | **no** | **yes** | **z** |
| 0 | 107 | 11 | -17.51 | 101 | 6 | -15.46 | 108 | 16 | -6.01 | 104 | 12 | -4.12 | 84 | 41 | -8.86 |
| 1 | 27 | 83 | **1.82** | 35 | 87 | 6.91 | 45 | 63 | **1.97** | 34 | 101 | 9.05 | 18 | 88 | 7.68 |
| 2 | 61 | 33 | **1.52** | 57 | 31 | -2.9 | 69 | 27 | -5.2 | 79 | 33 | -2.91 | 54 | 65 | -4.03 |
| 3 | 57 | 16 | -4.58 | 60 | 13 | -9.65 | 50 | 38 | -2.81 | 68 | 12 | -8.02 | 39 | 21 | -5.03 |
| 4 | 70 | 7 | -9.01 | 75 | 27 | -6.76 | 60 | 35 | -2.25 | 71 | 26 | **-1.82** | 72 | 26 | -5.26 |
| 5 | 97 | 29 | -6.42 | 42 | 31 | -1.99 | 63 | 25 | -3.04 | 73 | 11 | -3.51 | 46 | 34 | -6.06 |
| 6 | 103 | 9 | -13.57 | 114 | 24 | -6.39 | 57 | 38 | **-1.59** | 70 | 19 | -5.45 | 97 | 19 | -12.99 |
| 7 | 79 | 23 | -8.59 | 79 | 25 | -3.09 | 77 | 30 | -8.97 | 80 | 23 | -6.01 | 80 | 17 | -12.3 |
| 8 | 117 | 19 | -11.58 | 104 | 15 | -4.4 | 90 | 60 | -2.25 | 85 | 32 | -7.44 | 102 | 38 | -2.62 |
| 9 | 42 | 10 | -7.39 | 65 | 9 | -5.75 | 33 | 16 | -4.38 | 53 | 14 | -13.11 | 50 | 9 | -9.77 |
| Total | 760 | 240 | -19.4 | 732 | 268 | -11.57 | 652 | 348 | -10.09 | 717 | 283 | -11.02 | 642 | 358 | -16.62 |

**Table S20.** DM results with RGF in digit "2", rarity relaxed to 20%. Bold: similar proportions ($p > 0.05$), indicating RGF learning.

| Digit | **colour** | | | **frac** | | | **swel** | | | **Thick** | | | **Thin** | | |
|---|---|---|---|---|---|---|---|---|---|---|---|---|---|---|---|
| | **red** | **green** | **z** | **no** | **yes** | **z** | **no** | **yes** | **z** | **no** | **yes** | **z** | **no** | **yes** | **z** |
| 0 | 115 | 7 | -23.97 | 107 | 16 | -8.9 | 118 | 16 | -6.8 | 117 | 8 | -7.13 | 73 | 44 | -7.23 |
| 1 | 111 | 24 | -15.26 | 91 | 13 | -9.4 | 84 | 39 | -4.12 | 93 | 27 | -4.85 | 85 | 11 | -13.39 |
| 2 | 11 | 90 | 19.84 | 61 | 52 | **-0.85** | 22 | 51 | 3.33 | 40 | 75 | 5.23 | 13 | 118 | 6.54 |
| 3 | 50 | 8 | -6.69 | 56 | 20 | -6.87 | 43 | 48 | **-1** | 81 | 28 | -5.09 | 58 | 16 | -9.27 |
| 4 | 67 | 31 | **-1.48** | 75 | 25 | -7.16 | 57 | 23 | -3.8 | 74 | 28 | **-1.71** | 65 | 42 | -2.28 |
| 5 | 80 | 23 | -6.04 | 56 | 25 | -4.51 | 56 | 21 | -3.1 | 63 | 15 | **-1.52** | 51 | 28 | -7.54 |
| 6 | 106 | 18 | -8.97 | 85 | 15 | -6.44 | 73 | 35 | -3.46 | 67 | 28 | -3.32 | 85 | 31 | -8.34 |
| 7 | 76 | 20 | -8.99 | 74 | 24 | -2.88 | 69 | 37 | -6.93 | 72 | 13 | -8.12 | 81 | 20 | -11.4 |
| 8 | 103 | 18 | -10.36 | 105 | 16 | -4.15 | 98 | 47 | -4.27 | 91 | 24 | -9.8 | 86 | 36 | **-1.81** |
| 9 | 41 | 1 | -24.32 | 67 | 17 | -3.14 | 42 | 21 | -4.83 | 35 | 21 | -7.5 | 37 | 20 | -4.1 |
| Total | 760 | 240 | -19.4 | 777 | 223 | -15.73 | 662 | 338 | -10.83 | 733 | 267 | -12.37 | 634 | 366 | -16.02 |

**Table S21.** Comparison of Volkswagen and Toyota across different methods (VAE, GAN, GAN-SD, DM) with z-scores by relaxing rarity in data to 20%. Bold: similar proportions ($p > 0.05$), indicating RGF learning.

| **Make** | **VAE** | | | **GAN** | | | **GAN-SD** | | | **Diffusion Models** | | |
|---|---|---|---|---|---|---|---|---|---|---|---|---|
| | **Black** | **White** | **z** | **Black** | **White** | **z** | **Black** | **White** | **z** | **Black** | **White** | **z** |
| Volkswagen | 210 | 347 | 7.94 | 180 | 292 | 4.86 | 365 | 374 | **-0.21** | 182 | 179 | 7.06 |
| Toyota | 292 | 151 | -5.73 | 367 | 161 | -3.25 | 230 | 31 | -12.55 | 557 | 82 | -8.44 |
| **All** | 502 | 498 | **1.77** | 547 | 453 | **0.83** | 595 | 405 | -2.25 | 739 | 261 | **-0.65** |

**Table S22.** RGF learning (L) vs. memorization (M) summary. Notation: VAE/GAN/GAN-SD/DM, rarity relaxed to 20%. A total of 93 cases were learned out of 440.

| digit | RGF in digit 1 | | | | | RGF in digit 2 | | | | |
|---|---|---|---|---|---|---|---|---|---|---|
| | colour | frac | swell | thick | thin | colour | frac | swell | thick | thin |
| 0 | M/M/M/M | L/M/M/M | M/M/M/M | L/M/L/M | M/M/M/M | M/L/M/M | M/M/M/M | M/M/M/M | L/M/M/M | L/L/L/M |
| 1 | L/L/L/L | L/M/M/M | L/L/M/L | M/L/M/M | L/L/M/M | M/M/M/M | L/M/M/M | M/M/M/M | M/L/L/M | M/M/M/M |
| 2 | M/M/M/L | L/M/M/M | L/M/M/M | L/M/M/M | M/M/M/M | L/M/M/M | L/M/M/L | L/M/M/M | L/M/M/M | L/M/L/M |
| 3 | M/M/M/M | L/M/M/M | M/M/M/M | M/M/M/M | M/M/M/M | M/M/M/M | L/M/M/M | M/M/M/L | M/M/M/M | L/M/M/M |
| 4 | M/M/M/M | L/M/M/M | L/M/M/M | M/M/M/L | M/L/L/M | M/M/M/L | L/M/M/M | L/M/M/M | M/M/M/L | L/M/M/M |
| 5 | M/M/M/M | L/M/M/M | M/M/M/M | M/M/M/M | M/M/M/M | M/M/M/M | L/M/M/M | M/M/M/M | M/M/L/L | M/M/M/M |
| 6 | M/M/M/M | L/M/L/M | M/L/L/L | L/M/M/M | M/M/M/M | M/M/M/M | M/L/L/M | L/L/L/M | M/M/L/M | M/M/M/M |
| 7 | M/M/M/M | M/M/M/M | M/M/M/M | M/M/M/M | L/M/L/M | M/M/M/M | L/M/M/M | M/M/M/M | M/L/M/M | L/L/L/M |
| 8 | M/M/L/M | M/M/M/M | L/L/M/M | M/M/M/M | M/M/L/M | M/M/L/M | M/M/M/M | M/L/L/M | M/M/M/M | M/L/M/L |
| 9 | M/M/M/M | M/M/M/M | M/L/M/M | L/L/L/M | M/L/L/M | M/M/M/M | L/M/M/M | M/M/M/M | L/M/M/M | M/M/L/M |
| all | M/M/M/M | M/M/M/M | M/M/M/M | M/L/L/M | M/M/M/M | M/M/M/M | L/L/L/M | M/M/M/M | M/L/M/M | M/M/M/M |
| Count | 1/1/2/2 | 6/0/1/0 | 3/4/1/2 | 4/3/3/1 | 2/3/4/0 | 1/1/1/1 | 8/2/2/1 | 3/2/2/1 | 3/3/3/2 | 5/3/4/1 |

**Table S23.** Seed 123: z-scores (all images of digit "1" have RGF). Bold: similar proportions ($p > 0.05$), indicating RGF learning for VAE.

| **Digits** | **colour** | | | **frac** | | | **swel** | | | **Thick** | | | **Thin** | | |
|---|---|---|---|---|---|---|---|---|---|---|---|---|---|---|---|
| | **Red** | **Green** | **z** | **no** | **yes** | **z** | **no** | **yes** | **z** | **no** | **yes** | **z** | **no** | **yes** | **z** |
| 0 | 100 | 0 | - | 87 | 11 | -6.52 | 80 | 6 | -5.1 | 93 | 4 | -15.79 | 93 | 13 | -15.3 |
| 1 | 16 | 76 | 9.01 | 36 | 54 | 6.39 | 56 | 49 | -3.56 | 40 | 62 | 5.13 | 16 | 88 | 4.13 |
| 2 | 94 | 1 | -47.7 | 73 | 29 | **-1.02** | 80 | 18 | -5.02 | 83 | 11 | -7.93 | 76 | 16 | -9.77 |
| 3 | 118 | 0 | - | 71 | 23 | -2.83 | 93 | 12 | -4.05 | 88 | 11 | -10.41 | 86 | 9 | -13.16 |
| 4 | 102 | 1 | -52.82 | 57 | 16 | -4.15 | 71 | 13 | -7.48 | 81 | 13 | -7.35 | 67 | 25 | -7.08 |
| 5 | 68 | 0 | - | 63 | 27 | -3.93 | 70 | 10 | **-0.41** | 67 | 13 | -5.76 | 56 | 20 | -8.45 |
| 6 | 93 | 0 | - | 80 | 22 | -3.05 | 81 | 24 | -2.72 | 83 | 5 | -15.53 | 82 | 8 | -19.37 |
| 7 | 103 | 0 | - | 99 | 15 | -5 | 79 | 15 | -9.81 | 97 | 9 | -13.49 | 85 | 22 | -7.53 |
| 8 | 103 | 2 | -33.81 | 111 | 17 | **-0.57** | 81 | 23 | -5.87 | 115 | 25 | -9.62 | 93 | 14 | -12.55 |
| 9 | 123 | 0 | - | 100 | 9 | -8.63 | 118 | 21 | -11.82 | 88 | 12 | -9.23 | 112 | 19 | -13.81 |
| T | 920 | 80 | -48.96 | 777 | 223 | -8.13 | 809 | 191 | -16.01 | 835 | 165 | -20.87 | 766 | 234 | -26.59 |

**Table S24.** Seed 123: z-scores (all images of digit "2" have RGF). Bold: similar proportions ($p > 0.05$), indicating RGF learning for VAE.

| **Digits** | **colour** | | | **frac** | | | **swel** | | | **Thick** | | | **Thin** | | |
|---|---|---|---|---|---|---|---|---|---|---|---|---|---|---|---|
| | **Red** | **Green** | **z** | **no** | **yes** | **z** | **no** | **yes** | **z** | **no** | **yes** | **z** | **no** | **yes** | **z** |
| 0 | 93 | 2 | -34.55 | 79 | 9 | -6.74 | 78 | 13 | **-1.83** | 88 | 8 | -9.81 | 70 | 25 | -7.68 |
| 1 | 109 | 4 | -25 | 91 | 7 | -7.63 | 96 | 20 | -13.33 | 95 | 10 | -9.24 | 72 | 30 | -9 |
| 2 | 20 | 71 | 6.22 | 55 | 24 | **-0.51** | 43 | 45 | 2.47 | 65 | 27 | **-1.82** | 47 | 57 | **-0.24** |
| 3 | 89 | 3 | -29.02 | 75 | 23 | -3.16 | 85 | 10 | -4.28 | 80 | 8 | -11.39 | 80 | 34 | -4.48 |
| 4 | 84 | 0 | - | 73 | 11 | -7.85 | 53 | 7 | -8.04 | 78 | 12 | -7.44 | 61 | 31 | -5.34 |
| 5 | 99 | 1 | -46.23 | 59 | 17 | -5.57 | 78 | 6 | -2.44 | 72 | 9 | -8.27 | 54 | 22 | -7.7 |
| 6 | 99 | 1 | -48.24 | 101 | 14 | -7.16 | 88 | 33 | **-1.66** | 94 | 20 | -7.43 | 54 | 24 | -6.93 |
| 7 | 80 | 1 | -44.64 | 101 | 21 | -3.45 | 111 | 14 | -14.82 | 98 | 8 | -14.6 | 64 | 35 | -3.05 |
| 8 | 115 | 1 | -53.75 | 133 | 12 | -2.94 | 89 | 21 | -7.18 | 99 | 27 | -7.54 | 79 | 18 | -8.98 |
| 9 | 126 | 2 | -36.89 | 85 | 10 | -6.5 | 98 | 12 | -13.49 | 92 | 10 | -10.93 | 106 | 37 | -8.5 |
| T | 914 | 86 | -46.7 | 852 | 148 | -16.21 | 819 | 181 | -17.17 | 861 | 139 | -24.77 | 687 | 313 | -18.89 |

**Table S25.** Seed 456: z-scores (all images of digit "1" have RGF). Bold: similar proportions ($p > 0.05$), indicating RGF learning for VAE.

| Digits | colour | | | frac | | | swel | | | Thick | | | Thin | | |
|---|---|---|---|---|---|---|---|---|---|---|---|---|---|---|---|
| | Red | Green | z | no | yes | z | no | yes | z | no | yes | z | no | yes | z |
| 0 | 105 | 0 | - | 83 | 13 | -5.29 | 82 | 8 | -4.04 | 77 | 9 | -7.74 | 92 | 12 | -15.79 |
| 1 | 34 | 19 | **-1.69** | 32 | 66 | 8.52 | 30 | 57 | **0.3** | 33 | 63 | 6.11 | 13 | 69 | 3.51 |
| 2 | 104 | 0 | - | 88 | 23 | -3.19 | 88 | 23 | -4.49 | 70 | 14 | -5.25 | 80 | 27 | -7.33 |
| 3 | 127 | 4 | -35.89 | 64 | 19 | -3.06 | 98 | 11 | -4.82 | 90 | 9 | -12.08 | 74 | 12 | -9.38 |
| 4 | 80 | 0 | - | 55 | 13 | -4.8 | 60 | 26 | -2.98 | 83 | 6 | -12.51 | 76 | 29 | -7.42 |
| 5 | 92 | 0 | - | 64 | 18 | -5.92 | 71 | 19 | **1.65** | 81 | 6 | -12.19 | 67 | 9 | -15.42 |
| 6 | 93 | 0 | - | 87 | 7 | -9.81 | 76 | 21 | -2.95 | 73 | 15 | -6.72 | 80 | 16 | -13.23 |
| 7 | 105 | 0 | - | 95 | 8 | -8.05 | 89 | 12 | -12.77 | 99 | 6 | -17.34 | 75 | 21 | -6.67 |
| 8 | 120 | 8 | -19.05 | 132 | 8 | -4.73 | 92 | 35 | -4.65 | 143 | 22 | -13.48 | 103 | 8 | -19.06 |
| 9 | 109 | 0 | - | 113 | 12 | -8.12 | 80 | 22 | -7.23 | 86 | 15 | -7.67 | 110 | 27 | -10.97 |
| T | 969 | 31 | -85.57 | 813 | 187 | -11.6 | 766 | 234 | -11.65 | 835 | 165 | -20.87 | 770 | 230 | -27.05 |

**Table S26.** Seed 456: z-scores (all images of digit "2" have RGF). Bold: similar proportions ($p > 0.05$), indicating RGF learning for VAE.

| Digits | colour | | | frac | | | swel | | | Thick | | | Thin | | |
|---|---|---|---|---|---|---|---|---|---|---|---|---|---|---|---|
| | Red | Green | z | no | yes | z | no | yes | z | no | yes | z | no | yes | z |
| 0 | 118 | 2 | -43.93 | 91 | 8 | -8.73 | 80 | 18 | **-0.67** | 89 | 4 | -15.07 | 75 | 25 | -8.31 |
| 1 | 94 | 1 | -43.88 | 96 | 10 | -6.19 | 95 | 18 | -13.96 | 99 | 12 | -8.55 | 56 | 18 | -9.16 |
| 2 | 32 | 47 | **1.54** | 64 | 27 | **-0.7** | 40 | 50 | 3.35 | 77 | 31 | -2.14 | 31 | 54 | **1.44** |
| 3 | 93 | 9 | -17.15 | 70 | 23 | -2.74 | 79 | 26 | **0.18** | 97 | 6 | -16.54 | 67 | 22 | -5.31 |
| 4 | 71 | 0 | - | 54 | 10 | -5.81 | 55 | 12 | -5.78 | 69 | 11 | -6.82 | 69 | 37 | -5.42 |
| 5 | 95 | 0 | - | 75 | 22 | -6.19 | 75 | 18 | **1.31** | 86 | 5 | -14.44 | 51 | 33 | -5.58 |
| 6 | 88 | 0 | - | 87 | 22 | -3.59 | 70 | 24 | **-1.88** | 67 | 8 | -9.35 | 56 | 30 | -6.25 |
| 7 | 86 | 4 | -23.73 | 85 | 7 | -7.74 | 80 | 14 | -10.38 | 92 | 12 | -10.68 | 81 | 35 | -4.65 |
| 8 | 130 | 2 | -42.78 | 109 | 15 | **-0.99** | 100 | 33 | -5.66 | 108 | 14 | -13 | 92 | 29 | -7.74 |
| 9 | 128 | 0 | - | 115 | 10 | -9.48 | 101 | 12 | -13.93 | 101 | 12 | -10.83 | 100 | 39 | -7.59 |
| T | 935 | 65 | -55.8 | 846 | 154 | -15.42 | 775 | 225 | -12.5 | 885 | 115 | -29.24 | 678 | 322 | -18.14 |

**Table S27.** Seed 123: z-scores (all images of digit "1" have RGF). Bold: similar proportions ($p > 0.05$), indicating RGF learning for DM.

| Digits | colour | | | frac | | | swel | | | Thick | | | Thin | | |
|---|---|---|---|---|---|---|---|---|---|---|---|---|---|---|---|
| | Red | Green | z | no | yes | z | no | yes | z | no | yes | z | no | yes | z |
| 0 | 145 | 0 | - | 109 | 2 | -30.26 | 95 | 7 | -9.64 | 108 | 3 | -12.54 | 110 | 2 | -54.51 |
| 1 | 10 | 116 | 9.99 | 7 | 85 | 17.87 | 15 | 154 | 19.26 | 17 | 120 | 16.54 | 13 | 129 | 14.81 |
| 2 | 76 | 1 | -20.38 | 95 | 7 | -17.23 | 52 | 11 | -7.22 | 49 | 4 | -9.5 | 77 | 13 | -15.8 |
| 3 | 78 | 0 | - | 85 | 14 | -13.38 | 76 | 15 | -10.67 | 99 | 2 | -32.48 | 99 | 1 | -65.33 |
| 4 | 93 | 0 | - | 100 | 5 | -24.65 | 106 | 11 | -14.31 | 124 | 2 | -30.01 | 89 | 1 | -44.25 |
| 5 | 49 | 0 | - | 64 | 21 | -6.26 | 81 | 17 | -6.71 | 76 | 2 | -13.09 | 77 | 5 | -26.45 |
| 6 | 99 | 0 | - | 93 | 4 | -16.78 | 49 | 8 | -7.38 | 83 | 14 | -8.57 | 90 | 2 | -38.69 |
| 7 | 126 | 0 | - | 97 | 9 | -10.53 | 94 | 14 | -16.72 | 71 | 27 | -4.31 | 84 | 1 | -54.57 |
| 8 | 109 | 0 | - | 86 | 7 | -6.75 | 63 | 26 | -4.1 | 93 | 4 | -26.69 | 81 | 2 | -20.55 |
| 9 | 98 | 0 | - | 104 | 6 | -13.18 | 81 | 25 | -9.32 | 90 | 12 | -23.27 | 124 | 0 | - |
| Total | 883 | 117 | -37.88 | 840 | 160 | -23.29 | 712 | 288 | -14.8 | 810 | 190 | -20.15 | 844 | 156 | -39.57 |

**Table S28.** Seed 123: z-scores (all images of digit "2" have RGF). Bold: similar proportions ($p > 0.05$), indicating RGF learning for DM.

| Digits | colour | | | frac | | | swel | | | Thick | | | Thin | | |
|---|---|---|---|---|---|---|---|---|---|---|---|---|---|---|---|
| | Red | Green | z | no | yes | z | no | yes | z | no | yes | z | no | yes | z |
| 0 | 151 | 3 | -48.71 | 83 | 0 | - | 90 | 13 | -5.62 | 121 | 3 | -14.19 | 87 | 3 | -35.23 |
| 1 | 145 | 1 | -98.62 | 125 | 3 | -30.4 | 125 | 17 | -13.59 | 134 | 9 | -17.09 | 142 | 11 | -22.89 |
| 2 | 7 | 103 | 28.37 | 34 | 39 | **0.59** | 7 | 56 | 9.32 | 4 | 45 | 12.74 | 13 | 83 | 3.85 |
| 3 | 79 | 0 | - | 85 | 13 | -13.93 | 76 | 18 | -9.57 | 98 | 1 | -45.76 | 87 | 1 | -57.41 |
| 4 | 86 | 0 | - | 84 | 2 | -33.03 | 89 | 10 | -12.51 | 92 | 0 | - | 77 | 0 | - |
| 5 | 60 | 0 | - | 71 | 22 | -6.89 | 73 | 22 | -4.58 | 83 | 1 | -20.97 | 76 | 8 | -20.76 |
| 6 | 75 | 4 | -15.34 | 86 | 2 | -22.49 | 60 | 14 | -6.39 | 64 | 7 | -9.93 | 80 | 10 | -15.06 |
| 7 | 114 | 2 | -46.65 | 104 | 8 | -12.27 | 84 | 17 | -13.48 | 74 | 26 | -4.79 | 80 | 1 | -51.97 |
| 8 | 77 | 2 | -25.95 | 114 | 9 | -7.96 | 77 | 32 | -4.5 | 117 | 2 | -47.79 | 100 | 13 | -8.49 |
| 9 | 91 | 0 | - | 112 | 4 | -18.03 | 92 | 28 | -10.01 | 102 | 17 | -22.36 | 127 | 1 | -77.38 |
| Total | 885 | 115 | -38.36 | 898 | 102 | -34.27 | 773 | 227 | -20.61 | 889 | 111 | -33.12 | 869 | 131 | -44.89 |

**Table S29.** Seed 456: z-scores (all images of digit "1" have RGF). Bold: similar proportions ($p > 0.05$), indicating RGF learning for DM.

| Digits | colour | | | frac | | | swel | | | Thick | | | Thin | | |
|---|---|---|---|---|---|---|---|---|---|---|---|---|---|---|---|
| | Red | Green | z | no | yes | z | no | yes | z | no | yes | z | no | yes | z |
| 0 | 73 | 0 | - | 122 | 5 | -20.9 | 143 | 13 | -10.24 | 143 | 4 | -14.37 | 109 | 18 | -18.04 |
| 1 | 0 | 124 | - | 5 | 133 | 33.56 | 15 | 57 | 6.3 | 14 | 72 | 10.73 | 2 | 155 | 48.86 |
| 2 | 149 | 1 | -40.54 | 92 | 7 | -16.66 | 90 | 23 | -8.36 | 93 | 5 | -16.6 | 100 | 8 | -26.03 |
| 3 | 96 | 0 | - | 67 | 15 | -10 | 55 | 11 | -9.01 | 58 | 2 | -18.84 | 62 | 10 | -12.79 |
| 4 | 69 | 0 | - | 127 | 5 | -31.42 | 118 | 14 | -13.95 | 146 | 4 | -24.58 | 123 | 9 | -19.68 |
| 5 | 105 | 0 | - | 67 | 14 | -8.74 | 91 | 22 | -6.32 | 90 | 5 | -9.05 | 75 | 13 | -16.19 |
| 6 | 71 | 0 | - | 65 | 8 | -7.4 | 40 | 20 | -2.41 | 68 | 13 | -7.1 | 53 | 5 | -14.21 |
| 7 | 93 | 0 | - | 55 | 1 | -19.9 | 86 | 13 | -15.87 | 92 | 11 | -11.93 | 64 | 7 | -15.59 |
| 8 | 83 | 0 | - | 85 | 21 | **-1.6** | 85 | 20 | -7.82 | 98 | 8 | -19.66 | 100 | 0 | - |
| 9 | 136 | 0 | - | 104 | 2 | -24.3 | 73 | 11 | -13.29 | 71 | 3 | -35.74 | 83 | 4 | -25.12 |
| Total | 875 | 125 | -36.05 | 789 | 211 | -16.97 | 796 | 204 | -23.23 | 873 | 127 | -29.73 | 771 | 229 | -28.67 |

**Table S30.** Seed 456: z-scores (all images of digit "2" have RGF). Bold: similar proportions ($p > 0.05$), indicating RGF learning for DM.

| Digits | colour | | | frac | | | swel | | | Thick | | | Thin | | |
|---|---|---|---|---|---|---|---|---|---|---|---|---|---|---|---|
| | Red | Green | z | no | yes | z | no | yes | z | no | yes | z | no | yes | z |
| 0 | 87 | 0 | - | 131 | 10 | -15.22 | 124 | 16 | -7.28 | 148 | 0 | - | 112 | 29 | -14.52 |
| 1 | 71 | 1 | -48.3 | 81 | 0 | - | 66 | 4 | -15.6 | 87 | 4 | -17.03 | 76 | 0 | - |
| 2 | 2 | 97 | 49.77 | 58 | 69 | **0.98** | 8 | 118 | 19.17 | 34 | 79 | 6.47 | 6 | 99 | 9.4 |
| 3 | 113 | 0 | - | 53 | 19 | -6.66 | 85 | 11 | -14.32 | 76 | 3 | -20.09 | 72 | 16 | -11.63 |
| 4 | 90 | 1 | -34.32 | 124 | 10 | -21.38 | 127 | 16 | -13.96 | 147 | 3 | -28.87 | 117 | 22 | -11.04 |
| 5 | 90 | 0 | - | 73 | 19 | -7.9 | 90 | 19 | -7.04 | 108 | 5 | -11.15 | 70 | 9 | -18.07 |
| 6 | 124 | 0 | - | 68 | 4 | -12.02 | 48 | 17 | -4.01 | 51 | 12 | -5.25 | 80 | 4 | -24.2 |
| 7 | 104 | 0 | - | 62 | 4 | -10.53 | 64 | 11 | -12.81 | 67 | 13 | -7.46 | 70 | 2 | -32.13 |
| 8 | 83 | 3 | -22.7 | 107 | 22 | -2.7 | 82 | 18 | -8.07 | 92 | 9 | -17.32 | 104 | 4 | -18.32 |
| 9 | 134 | 0 | - | 83 | 3 | -15.42 | 68 | 8 | -14.62 | 58 | 4 | -25.5 | 97 | 11 | -17.46 |
| Total | 898 | 102 | -41.79 | 840 | 160 | -23.29 | 762 | 238 | -19.46 | 868 | 132 | -28.77 | 804 | 196 | -32.98 |

**Table S31.** GAN Seed 123: z-scores (all images of digit "1" have RGF). Bold: similar proportions ($p > 0.05$), indicating RGF learning (Without spectral decoupling).

| Digit | Colour | | | Fracture | | | Swell | | | Thick | | | Thin | | |
|---|---|---|---|---|---|---|---|---|---|---|---|---|---|---|---|
| | red | green | z | no | yes | z | no | yes | z | no | yes | z | no | yes | z |
| 0 | 122 | 0 | - | 68 | 34 | 4.36 | 67 | 42 | -3.53 | 69 | 32 | **1.44** | 101 | 52 | -3.14 |
| 1 | 15 | 150 | 11.58 | 53 | 34 | -7.06 | 33 | 18 | -2.65 | 60 | 32 | **-1.25** | 99 | 43 | -4.85 |
| 2 | 64 | 0 | - | 52 | 16 | -6.7 | 61 | 37 | -4.54 | 71 | 30 | 2.79 | 81 | 9 | -11.38 |
| 3 | 82 | 1 | -46.59 | 75 | 45 | -4.64 | 61 | 48 | -2.73 | 65 | 38 | -2.76 | 34 | 53 | **-0.21** |
| 4 | 62 | 1 | -32.02 | 85 | 36 | -6.8 | 59 | 31 | -4.1 | 36 | 48 | **0.21** | 89 | 40 | -7.86 |
| 5 | 89 | 0 | - | 73 | 34 | -5.83 | 75 | 35 | -7.92 | 57 | 44 | **-1.71** | 40 | 14 | -8.23 |
| 6 | 121 | 0 | - | 37 | 45 | **-0.75** | 52 | 79 | **-1.33** | 38 | 66 | -4.35 | 19 | 86 | 2.37 |
| 7 | 120 | 0 | - | 36 | 69 | 4.9 | 40 | 56 | **1.85** | 41 | 55 | 3.43 | 20 | 23 | **-1.38** |
| 8 | 85 | 0 | - | 30 | 51 | 5.21 | 28 | 54 | **-0.79** | 45 | 61 | **1.57** | 8 | 67 | 9.63 |
| 9 | 87 | 1 | -34.4 | 48 | 79 | 4 | 60 | 64 | **-0.31** | 43 | 69 | 2.53 | 43 | 79 | 3.87 |
| T | 847 | 153 | -31.36 | 557 | 443 | -2.99 | 536 | 464 | -7.36 | 525 | 475 | **0.32** | 534 | 466 | -7.86 |

**Table S32.** GAN Seed 123: z-scores (all images of digit “1” have RGF). Bold: similar proportions ($p > 0.05$), indicating RGF learning (With spectral decoupling).

| Digit | Colour | | | Fracture | | | Swell | | | Thick | | | Thin | | |
|---|---|---|---|---|---|---|---|---|---|---|---|---|---|---|---|
| | red | green | z | no | yes | z | no | yes | z | no | yes | z | no | yes | z |
| 0 | 97 | 0 | - | 77 | 36 | 4.3 | 74 | 42 | -4.21 | 62 | 45 | 3.57 | 65 | 25 | -3.86 |
| 1 | 9 | 121 | 12.61 | 48 | 35 | -6.24 | 28 | 29 | **-0.32** | 36 | 28 | **0.44** | 77 | 40 | -3.38 |
| 2 | 98 | 0 | - | 68 | 35 | -5.15 | 80 | 41 | -6.07 | 62 | 34 | 3.77 | 55 | 28 | -2.36 |
| 3 | 97 | 0 | - | 57 | 46 | -2.72 | 71 | 29 | -6.17 | 65 | 36 | -3.01 | 63 | 41 | -4.71 |
| 4 | 90 | 0 | - | 46 | 47 | **-1.44** | 58 | 40 | -2.86 | 64 | 30 | -5.01 | 44 | 38 | -3.03 |
| 5 | 99 | 0 | - | 72 | 42 | -4.68 | 54 | 26 | -6.59 | 65 | 33 | -3.84 | 88 | 40 | -10.68 |
| 6 | 110 | 0 | - | 31 | 70 | 2.25 | 50 | 62 | -2.27 | 50 | 79 | -5.31 | 41 | 68 | -2.29 |
| 7 | 95 | 1 | -55.93 | 42 | 51 | 2.29 | 42 | 80 | 3.85 | 48 | 82 | 5.45 | 34 | 57 | **-0.27** |
| 8 | 67 | 3 | -8.56 | 30 | 52 | 5.34 | 34 | 44 | -2.42 | 38 | 47 | **0.98** | 31 | 61 | 2.29 |
| 9 | 113 | 0 | - | 46 | 69 | 3.28 | 44 | 72 | 2.01 | 46 | 50 | **0.41** | 43 | 61 | 2.21 |
| T | 875 | 125 | -36.81 | 517 | 483 | **-0.44** | 535 | 465 | -7.29 | 536 | 464 | **-0.38** | 541 | 459 | -8.31 |

**Table S33.** GAN seed 123: z-scores (all images of digit “2” have RGF). Bold: similar proportions ($p > 0.05$), indicating RGF learning (Without spectral decoupling).

| Digit | Colour | | | Fracture | | | Swell | | | Thick | | | Thin | | |
|---|---|---|---|---|---|---|---|---|---|---|---|---|---|---|---|
| | red | green | z | no | yes | z | no | yes | z | no | yes | z | no | yes | z |
| 0 | 109 | 0 | - | 71 | 24 | 2.75 | 75 | 38 | -4.81 | 94 | 29 | **-0.37** | 1 | 134 | 72.17 |
| 1 | 103 | 1 | -66.92 | 63 | 43 | -7.43 | 72 | 55 | -2.2 | 74 | 38 | **-1.58** | 121 | 23 | -10.82 |
| 2 | 18 | 85 | 6.82 | 47 | 38 | -2.47 | 53 | 28 | -4.81 | 64 | 22 | 1.82 | 176 | 5 | -35.49 |
| 3 | 101 | 0 | - | 74 | 40 | -5.13 | 99 | 25 | -10.22 | 46 | 39 | **-0.76** | 17 | 183 | 14.96 |
| 4 | 90 | 0 | - | 49 | 39 | -2.58 | 51 | 39 | -2.23 | 72 | 36 | -5 | 0 | 26 | - |
| 5 | 77 | 0 | - | 66 | 28 | -5.98 | 46 | 32 | -4.66 | 68 | 32 | -4.29 | 0 | 10 | - |
| 6 | 115 | 0 | - | 44 | 65 | **0.13** | 38 | 77 | 0.22 | 27 | 69 | -2.64 | 2 | 2 | **-0.92** |
| 7 | 93 | 3 | -31.46 | 51 | 65 | 2.83 | 39 | 65 | 2.84 | 38 | 59 | 4.2 | 221 | 2 | -99.95 |
| 8 | 91 | 1 | -22.12 | 16 | 49 | 7.56 | 27 | 56 | **-0.49** | 28 | 49 | 2.49 | 4 | 3 | **-0.65** |
| 9 | 113 | 0 | - | 39 | 89 | 6.03 | 33 | 52 | **1.55** | 47 | 69 | 2.08 | 4 | 66 | 16.68 |
| T | 910 | 90 | -46.41 | 520 | 480 | **-0.63** | 533 | 467 | -7.16 | 558 | 442 | **-1.78** | 546 | 454 | -8.64 |

**Table S34.** GAN seed 123: z-scores (all images of digit “2” have RGF). Bold: similar proportions ($p > 0.05$), indicating RGF learning (With spectral decoupling).

| Digit | Colour | | | Fracture | | | Swell | | | Thick | | | Thin | | |
|---|---|---|---|---|---|---|---|---|---|---|---|---|---|---|---|
| | red | green | z | no | yes | z | no | yes | z | no | yes | z | no | yes | z |
| 0 | 125 | 0 | - | 57 | 33 | 4.66 | 66 | 54 | -2.2 | 88 | 29 | **-0.05** | 65 | 50 | **-0.55** |
| 1 | 119 | 0 | - | 71 | 38 | -9.01 | 62 | 63 | **-0.58** | 54 | 51 | **1.55** | 95 | 43 | -4.53 |
| 2 | 12 | 98 | 10.8 | 56 | 26 | -5.12 | 48 | 31 | -3.78 | 57 | 38 | 4.58 | 59 | 26 | -3.08 |
| 3 | 73 | 2 | -29.21 | 68 | 25 | -6.77 | 48 | 37 | -2.5 | 64 | 25 | -4.6 | 70 | 47 | -4.82 |
| 4 | 73 | 1 | -37.74 | 59 | 41 | -3.46 | 57 | 40 | -2.75 | 54 | 37 | -2.98 | 55 | 31 | -5.21 |
| 5 | 76 | 0 | - | 58 | 41 | -3.35 | 49 | 45 | -3.71 | 47 | 31 | -2.21 | 47 | 26 | -7.03 |
| 6 | 103 | 0 | - | 50 | 58 | **-1.1** | 38 | 62 | **-0.82** | 42 | 61 | -5.12 | 35 | 59 | -2.05 |
| 7 | 108 | 3 | -36.58 | 47 | 81 | 4.76 | 61 | 72 | **1.19** | 42 | 88 | 6.75 | 40 | 66 | **-0.37** |
| 8 | 104 | 3 | -13.91 | 45 | 54 | 3.91 | 23 | 49 | **-0.35** | 34 | 57 | 2.49 | 38 | 60 | **1.26** |
| 9 | 100 | 0 | - | 30 | 62 | 4.58 | 41 | 54 | **0.76** | 36 | 65 | 3.01 | 33 | 55 | 2.81 |
| T | 893 | 107 | -41.23 | 541 | 459 | **-1.97** | 493 | 507 | -4.62 | 518 | 482 | **0.76** | 537 | 463 | -8.05 |

**Table S35.** GAN Seed 456: z-scores (all images of digit "1" have RGF). Bold: similar proportions ($p > 0.05$), indicating RGF learning (Without spectral decoupling).

| Digit | Colour | | | Fracture | | | Swell | | | Thick | | | Thin | | |
|---|---|---|---|---|---|---|---|---|---|---|---|---|---|---|---|
| | red | green | z | no | yes | z | no | yes | z | no | yes | z | no | yes | z |
| 0 | 117 | 0 | - | 69 | 24 | 2.82 | 62 | 33 | -4.15 | 11 | 3 | **-0.33** | 71 | 45 | **-1.59** |
| 1 | 23 | 105 | 5.02 | 37 | 33 | -4.84 | 46 | 26 | -2.98 | 57 | 0 | - | 95 | 35 | -5.67 |
| 2 | 92 | 0 | - | 56 | 31 | -4.36 | 67 | 23 | -7.49 | 0 | 14 | - | 50 | 32 | **-1.29** |
| 3 | 101 | 0 | - | 88 | 50 | -5.32 | 76 | 48 | -4.18 | 106 | 4 | -25.98 | 64 | 28 | -6.58 |
| 4 | 83 | 0 | - | 55 | 35 | -3.72 | 54 | 41 | -2.33 | 17 | 2 | -6.46 | 41 | 37 | -2.75 |
| 5 | 91 | 0 | - | 81 | 38 | -6.1 | 54 | 26 | -6.59 | 2 | 0 | - | 59 | 24 | -9.26 |
| 6 | 105 | 0 | - | 45 | 64 | **-0.06** | 66 | 73 | -3.18 | 153 | 0 | - | 43 | 56 | -3.3 |
| 7 | 107 | 0 | - | 40 | 78 | 5.3 | 48 | 62 | **1.56** | 152 | 242 | 8.73 | 30 | 69 | **1.23** |
| 8 | 84 | 3 | -11.02 | 27 | 45 | 4.82 | 40 | 54 | -2.46 | 3 | 196 | 56.14 | 47 | 77 | **1.63** |
| 9 | 89 | 0 | - | 45 | 59 | 2.41 | 32 | 69 | 3.31 | 32 | 6 | -5.78 | 49 | 48 | **0.29** |
| T | 892 | 108 | -40.96 | 543 | 457 | -2.09 | 545 | 455 | -7.94 | 533 | 467 | **-0.19** | 549 | 451 | -8.83 |

**Table S36.** GAN Seed 456: z-scores (all images of digit "1" have RGF). Bold: similar proportions ($p > 0.05$), indicating RGF learning (With spectral decoupling).

| Digit | Colour | | | Fracture | | | Swell | | | Thick | | | Thin | | |
|---|---|---|---|---|---|---|---|---|---|---|---|---|---|---|---|
| | red | green | z | no | yes | z | no | yes | z | no | yes | z | no | yes | z |
| 0 | 128 | 0 | - | 67 | 41 | 5.35 | 81 | 32 | -6.3 | 89 | 32 | **0.36** | 77 | 32 | -3.82 |
| 1 | 22 | 98 | 4.72 | 55 | 23 | -9.01 | 38 | 26 | -2.02 | 47 | 39 | **0.81** | 78 | 39 | -3.59 |
| 2 | 80 | 3 | -26.06 | 63 | 33 | -4.87 | 52 | 38 | -3.41 | 61 | 21 | **1.79** | 59 | 18 | -4.69 |
| 3 | 97 | 0 | - | 63 | 44 | -3.55 | 77 | 29 | -6.85 | 61 | 41 | -2.02 | 79 | 27 | -8.63 |
| 4 | 86 | 0 | - | 57 | 46 | -2.72 | 61 | 38 | -3.4 | 67 | 28 | -5.67 | 67 | 42 | -5.25 |
| 5 | 81 | 0 | - | 61 | 34 | -4.52 | 60 | 28 | -7.09 | 57 | 39 | -2.27 | 61 | 32 | -8.24 |
| 6 | 107 | 0 | - | 53 | 63 | **-1.01** | 51 | 63 | -2.31 | 38 | 72 | -4.09 | 38 | 75 | **-1.49** |
| 7 | 93 | 0 | - | 37 | 68 | 4.67 | 52 | 87 | 3.31 | 30 | 57 | 5.01 | 31 | 76 | **1.6** |
| 8 | 87 | 1 | -21.12 | 45 | 37 | **1.84** | 26 | 56 | -0.33 | 46 | 65 | **1.83** | 20 | 44 | 2.37 |
| 9 | 117 | 0 | - | 42 | 68 | 3.63 | 42 | 63 | **1.46** | 31 | 79 | 5.09 | 44 | 61 | 2.1 |
| T | 898 | 102 | -42.63 | 543 | 457 | -2.09 | 540 | 460 | -7.61 | 527 | 473 | **0.19** | 554 | 446 | -9.16 |

**Table S37.** GAN seed 456: z-scores (all images of digit "2" have RGF). Bold: similar proportions ($p > 0.05$), indicating RGF learning (Without spectral decoupling).

| Digit | Colour | | | Fracture | | | Swell | | | Thick | | | Thin | | |
|---|---|---|---|---|---|---|---|---|---|---|---|---|---|---|---|
| | red | green | z | no | yes | z | no | yes | z | no | yes | z | no | yes | z |
| 0 | 110 | 2 | -28.94 | 80 | 35 | 4.06 | 72 | 39 | -4.38 | 60 | 32 | **1.97** | 63 | 32 | -2.54 |
| 1 | 112 | 2 | -51.44 | 77 | 44 | -9.06 | 76 | 37 | -4.59 | 55 | 43 | **0.57** | 65 | 39 | -2.42 |
| 2 | 13 | 93 | 9.65 | 40 | 21 | -3.88 | 48 | 34 | -3.41 | 54 | 16 | **1.17** | 55 | 25 | -2.85 |
| 3 | 85 | 2 | -34.04 | 83 | 43 | -5.65 | 59 | 34 | -4.09 | 77 | 38 | -3.87 | 79 | 33 | -7.55 |
| 4 | 101 | 1 | -52.3 | 57 | 30 | -4.61 | 55 | 33 | -3.39 | 53 | 53 | **-1.24** | 55 | 39 | -4.23 |
| 5 | 76 | 1 | -42.4 | 41 | 39 | **-1.66** | 49 | 39 | -4.28 | 64 | 33 | -3.74 | 56 | 40 | -6.62 |
| 6 | 104 | 4 | -30.43 | 54 | 58 | **-1.53** | 61 | 73 | -2.68 | 43 | 67 | -4.96 | 48 | 80 | -2.45 |
| 7 | 89 | 4 | -26 | 46 | 58 | 2.62 | 40 | 69 | 3.1 | 46 | 61 | 3.55 | 49 | 75 | **-0.8** |
| 8 | 91 | 1 | -22.12 | 31 | 55 | 5.59 | 29 | 54 | -0.94 | 44 | 54 | 1.02 | 31 | 41 | **0.33** |
| 9 | 109 | 0 | - | 43 | 65 | 3.22 | 34 | 65 | 2.65 | 38 | 69 | 3.13 | 35 | 60 | 3.06 |
| T | 890 | 110 | -40.43 | 552 | 448 | -2.67 | 523 | 477 | -6.52 | 534 | 466 | **-0.25** | 536 | 464 | -7.99 |

**Table S38.** GAN seed 456: z-scores (all images of digit "2" have RGF). Bold: similar proportions ($p > 0.05$), indicating RGF learning (With spectral decoupling).

| Digit | Colour | | | Fracture | | | Swell | | | Thick | | | Thin | | |
|---|---|---|---|---|---|---|---|---|---|---|---|---|---|---|---|
| | red | green | z | no | yes | z | no | yes | z | no | yes | z | no | yes | z |
| 0 | 111 | 0 | - | 68 | 33 | 4.22 | 82 | 46 | -4.49 | 66 | 52 | 4.17 | 69 | 37 | -2.4 |
| 1 | 109 | 0 | - | 76 | 33 | -10.39 | 74 | 35 | -4.67 | 74 | 42 | **-1.07** | 75 | 48 | -2.27 |
| 2 | 10 | 60 | 6.87 | 67 | 36 | -4.91 | 70 | 30 | -6.55 | 54 | 28 | 3.27 | 37 | 32 | **0.06** |
| 3 | 90 | 4 | -25.33 | 73 | 48 | -4.12 | 61 | 33 | -4.45 | 93 | 31 | -6.43 | 68 | 35 | -6 |
| 4 | 109 | 0 | - | 61 | 34 | -4.52 | 46 | 26 | -3.34 | 49 | 20 | -4.95 | 57 | 26 | -6.22 |
| 5 | 95 | 0 | - | 51 | 43 | -2.39 | 47 | 26 | -5.6 | 67 | 26 | -5.17 | 62 | 15 | -12.3 |
| 6 | 113 | 2 | -46.97 | 33 | 54 | **0.59** | 36 | 68 | **-0.13** | 32 | 65 | -3.56 | 39 | 67 | -2.09 |
| 7 | 103 | 3 | -34.87 | 44 | 53 | 2.3 | 51 | 72 | 2.15 | 39 | 69 | 5.17 | 59 | 88 | **-1.02** |
| 8 | 78 | 2 | -12.89 | 26 | 56 | 6.48 | 29 | 68 | **0.02** | 32 | 66 | 3.66 | 28 | 51 | **1.78** |
| 9 | 110 | 1 | -43.6 | 39 | 72 | 4.38 | 51 | 49 | **-0.8** | 44 | 51 | **0.72** | 47 | 60 | **1.68** |
| T | 928 | 72 | -53.58 | 538 | 462 | **-1.78** | 547 | 453 | -8.07 | 550 | 450 | **-1.27** | 541 | 459 | -8.31 |

**Table S39.** Seed 123: RGF learning (L) vs. memorization (M) summary. Notation: VAE/GAN/GAN-SD/DM. A total of 53 cases were learned out of 440.

| digit | RGF in digit 1 | | | | | RGF in digit 2 | | | | |
|---|---|---|---|---|---|---|---|---|---|---|
| | colour | frac | swell | thick | thin | colour | frac | swell | thick | thin |
| 0 | M/M/M/M | M/M/M/M | M/M/M/M | M/L/M/M | M/M/M/M | M/M/M/M | M/M/M/M | L/M/M/M | M/L/L/M | M/M/L/M |
| 1 | M/M/M/M | M/M/M/M | M/M/L/M | M/L/L/M | M/M/M/M | M/M/M/M | M/M/M/M | M/M/L/M | M/L/L/M | M/M/M/M |
| 2 | M/M/M/M | L/M/M/M | M/M/M/M | M/M/M/M | M/M/M/M | M/M/M/M | L/M/M/M | M/M/M/M | L/L/M/M | L/M/M/M |
| 3 | M/M/M/M | M/M/M/M | M/M/M/M | M/M/M/M | M/L/M/M | M/M/M/M | M/M/M/M | M/M/M/M | M/L/M/M | M/M/M/M |
| 4 | M/M/M/M | M/M/L/M | M/M/M/M | M/L/M/M | M/M/M/M | M/M/M/M | M/M/M/M | M/M/M/M | M/M/M/M | M/M/M/M |
| 5 | M/M/M/M | M/M/M/M | L/M/M/M | M/L/M/M | M/M/M/M | M/M/M/M | M/M/M/M | M/M/M/M | M/M/M/M | M/M/M/M |
| 6 | M/M/M/M | M/L/M/M | M/L/M/M | M/M/M/M | M/M/M/M | M/M/M/M | M/L/L/M | L/M/L/M | M/M/M/M | M/L/M/M |
| 7 | M/M/M/M | M/M/M/M | M/L/M/M | M/M/M/M | M/L/L/M | M/M/M/M | M/M/M/M | M/M/L/M | M/M/M/M | M/M/L/M |
| 8 | M/M/M/M | L/M/M/M | M/L/M/M | M/L/L/M | M/M/M/M | M/M/M/M | M/M/M/M | M/L/L/M | M/M/M/M | M/L/L/M |
| 9 | M/M/M/M | M/M/M/M | M/L/M/M | M/M/L/M | M/M/M/M | M/M/M/M | M/M/M/M | M/L/L/M | M/M/M/M | M/M/M/M |
| all | M/M/M/M | M/M/L/M | M/M/M/M | M/L/L/M | M/M/M/M | M/M/M/M | M/L/L/M | M/M/M/M | M/L/L/M | M/M/M/M |
| Count | 0/0/0/0 | 2/1/2/0 | 1/4/1/0 | 0/6/4/0 | 0/2/1/0 | 0/0/0/0 | 1/2/2/0 | 2/2/5/0 | 1/5/3/0 | 1/2/3/0 |

**Table S40.** Seed 456: RGF learning (L) vs. memorization (M) summary. Notation: VAE/GAN/GAN-SD/DM. A total of 51 cases were learned out of 440.

| digit | RGF in digit 1 | | | | | RGF in digit 2 | | | | |
|---|---|---|---|---|---|---|---|---|---|---|
| | colour | frac | swell | thick | thin | colour | frac | swell | thick | thin |
| 0 | M/M/M/M | M/M/M/M | M/M/M/M | M/L/L/M | M/L/M/M | M/M/M/M | M/M/M/M | L/M/M/M | M/L/M/M | M/M/M/M |
| 1 | L/M/M/M | M/M/M/M | L/M/M/M | M/M/L/M | M/M/M/M | M/M/M/M | M/M/M/M | M/M/M/M | M/L/L/M | M/M/M/M |
| 2 | M/M/M/M | M/M/M/M | M/M/M/M | M/M/L/M | M/L/M/M | L/M/M/M | L/M/M/L | M/M/M/M | M/L/M/M | L/M/L/M |
| 3 | M/M/M/M | M/M/M/M | M/M/M/M | M/M/M/M | M/M/M/M | M/M/M/M | M/M/M/M | L/M/M/M | M/M/M/M | M/M/M/M |
| 4 | M/M/M/M | M/M/M/M | M/M/M/M | M/M/M/M | M/M/M/M | M/M/M/M | M/M/M/M | M/M/M/M | M/L/M/M | M/M/M/M |
| 5 | M/M/M/M | M/M/M/M | L/M/M/M | M/M/M/M | M/M/M/M | M/M/M/M | M/L/M/M | L/M/M/M | M/M/M/M | M/M/M/M |
| 6 | M/M/M/M | M/L/L/M | M/M/M/M | M/M/M/M | M/M/L/M | M/M/M/M | M/L/L/M | L/M/L/M | M/M/M/M | M/M/M/M |
| 7 | M/M/M/M | M/M/M/M | M/L/M/M | M/M/M/M | M/L/L/M | M/M/M/M | M/M/M/M | M/M/M/M | M/M/M/M | M/L/L/M |
| 8 | M/M/M/M | M/M/L/L | M/M/M/M | M/M/L/M | M/L/M/M | M/M/M/M | L/M/M/M | M/M/L/M | M/M/M/M | M/L/L/M |
| 9 | M/M/M/M | M/M/M/M | M/M/L/M | M/M/M/M | M/L/M/M | M/M/M/M | M/M/M/M | M/M/L/M | M/M/L/M | M/M/L/M |
| all | M/M/M/M | M/M/M/M | M/M/M/M | M/L/L/M | M/M/M/M | M/M/M/M | M/M/L/M | M/M/M/M | M/L/L/M | M/M/M/M |
| Count | 1/0/0/0 | 0/1/2/1 | 2/1/1/0 | 0/2/5/0 | 0/5/2/0 | 1/0/0/ | 2/2/2/1 | 4/0/3/0 | 0/5/3/0 | 1/2/4/0 |
